# clonealign: statistical integration of independent single-cell RNA and DNA sequencing data from human cancers

**DOI:** 10.1186/s13059-019-1645-z

**Published:** 2019-03-12

**Authors:** Kieran R. Campbell, Adi Steif, Emma Laks, Hans Zahn, Daniel Lai, Andrew McPherson, Hossein Farahani, Farhia Kabeer, Ciara O’Flanagan, Justina Biele, Jazmine Brimhall, Beixi Wang, Pascale Walters, IMAXT Consortium, Alexandre Bouchard-Côté, Samuel Aparicio, Sohrab P. Shah

**Affiliations:** 1 Department of Molecular Oncology, British Columbia Cancer Research Centre, Vancouver, British Columbia, Canada; 2 Department of Statistics, University of British Columbia, Vancouver, British Columbia, Canada; 3 UBC Data Science Institute, University of British Columbia, Vancouver, British Columbia, Canada; 4 Genome Science and Technology Graduate Program, University of British Columbia, Vancouver, British Columbia, Canada; 5 Computational Oncology, Dept. of Epidemiology and Biostatistics, Memorial Sloan Kettering Cancer Center, New York, NY USA; 6 Department of Pathology and Laboratory Medicine, University of British Columbia, Vancouver, British Columbia, Canada; 7 Michael Smith Laboratories, University of British Columbia, Vancouver, British Columbia, Canada; 8 CRUK IMAXT Grand Challenge Consortium, Cambridge, UK

## Abstract

Measuring gene expression of tumor clones at single-cell resolution links functional consequences to somatic alterations. Without scalable methods to simultaneously assay DNA and RNA from the same single cell, parallel single-cell DNA and RNA measurements from independent cell populations must be mapped for genome-transcriptome association. We present clonealign, which assigns gene expression states to cancer clones using single-cell RNA and DNA sequencing independently sampled from a heterogeneous population. We apply clonealign to triple-negative breast cancer patient-derived xenografts and high-grade serous ovarian cancer cell lines and discover clone-specific dysregulated biological pathways not visible using either sequencing method alone.

**Electronic supplementary material:**

The online version of this article (10.1186/s13059-019-1645-z) contains supplementary material, which is available to authorized users.

## Background

Recent advances in genomic measurement technologies have allowed for unprecedented scalable interrogation of the genomes and transcriptomes of single cells [1, 2]. Such technologies are of particular interest in cancer, enabling measurement of cell-autonomous properties which constitute tumors as a whole. Molecular phenotyping at the single-cell level enables reconstruction of tumor life histories through phylogenetic analysis [3, 4], assessment of cell types in the tumor microenvironment [5], and quantification of intra-tumoral heterogeneity and its clinical implications [6, 7].

Theoretically, combined assays sequencing both RNA and DNA from the same single cell will provide a measurement of genomic alterations impacting transcriptional programs. This would yield a powerful single-cell level genotype-phenotype read out, encoding relevant malignant properties of clonal expansion, proliferation, and metastasis. Moreover, drug responses in cancer are commonly driven by positive and negative evolutionary selection of mutation-induced phenotypes, but genome-independent responses via dynamic epigenetic re-wiring of transcriptional programs have also been observed [8]. Thus, multimodal approaches assaying both DNA and RNA are essential for comprehensive study of drug response.

While pioneering technologies such as G&T-seq [9] and DR-seq [10] sequence both the DNA and RNA from single cells, they measure few cells compared to assays that sequence DNA or RNA alone such as Direct Library Preparation (DLP [1]) or 10X genomics single-cell RNA-seq [2], and thus provide only a limited view of each tumor’s genomic and transcriptional heterogeneity. However, independently sampled single-cell measurements introduce a new analytical challenge of how to associate cells across each modality. Assuming a population structure with a fixed number of clones, this can be expressed as a mapping problem, whereby cells measured with transcriptome assays must be aligned to those measured with a genome assay.

## Results

**Fig. 1:**
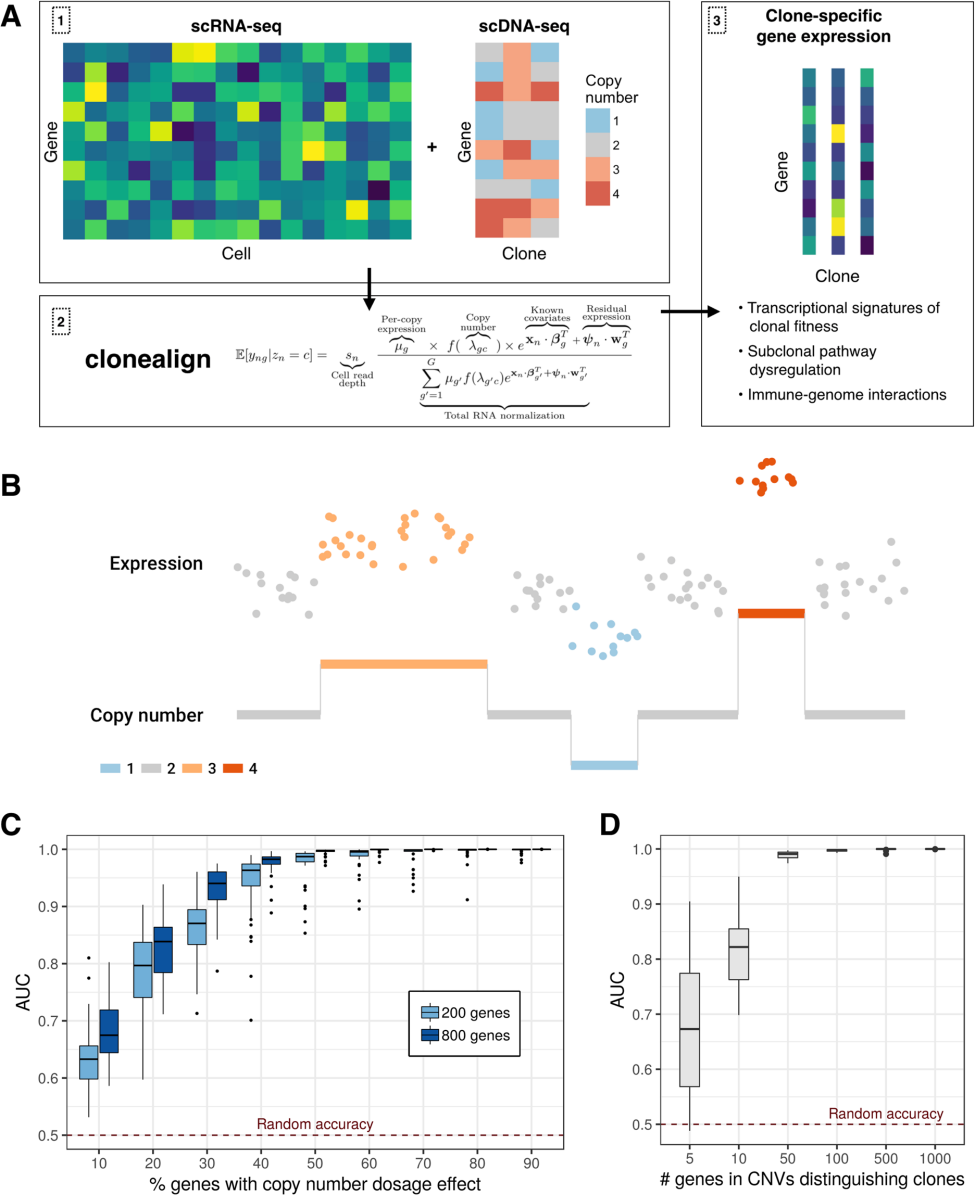
Assigning single-cell RNA-seq to clone-of-origin using clonealign. **a** Given independently sampled single-cell DNA- and RNA-seq from the same tumor, the clonealign statistical framework assigns each cell’s gene expression profile to its clone-of-origin, uncovering transcriptional signatures of clonal fitness. **b** To relate cells as measured in RNA-space to their clones measured in DNA-space, we assume a relationship between gene copy number and gene expression (simulated data). **c** Simulations demonstrate the robustness of clonealign to the underlying proportion of genes exhibiting a copy number dosage effect. Even if only 30% of genes have a clone-specific copy number effect on expression, clones can still be accurately assigned with an average AUC >0.8. **d** Simulations demonstrate clonal assignment is accurate even when as few as 10–50 genes lie in regions of differing copy number between clones, allowing clonal assignment from only small-scale genomic rearrangements

To address this mapping problem we introduce clonealign, a statistical method to assign cells measured with single-cell RNA-seq to clones derived from low-coverage single-cell DNA-seq (Fig. 1a).

In our approach, we assume clones are defined through grouped cell subsets which share to a first approximation similar genomic copy number structure (e.g., through phylogenetic reconstruction or dimensionality reduction [11]). In order to relate the independent measurements, we assume that an increase in the copy number of a gene will result in a corresponding increase in that gene’s expression and vice versa (Fig. 1b), a relationship previously observed in joint RNA-DNA assays in bulk tissues [12] and at the single-cell level [9, 10, 13].

Based on this relationship, we formulate a statistical model that explains the observed gene expression pattern in terms of the copy number profile of a clone present in the scDNA-seq data and thus assigns each cell to a clone (see the “Methods” section). Furthermore, clonealign can integrate the additional information given by alleleic imbalance in expression caused by clone-specific loss-of-heterozygosity (LOH) events when such data is available (see the “Methods” section).

To test the robustness of the clonealign model, we performed comprehensive simulations (see the “Methods” section) across a wide range of scenarios. We began by simulating datasets where a certain proportion of genes have no CN-expression relationship and clone assignments re-inferred using clonealign assuming all genes had CN-dependent expression. We found that clonealign is highly robust to variation in the underlying proportion of genes with CN-dependent expression (Fig. 1c), with a median area under the receiver-operator curve (AUC) greater than 0.8 even when only 30% of genes have such a dosage effect. We next examined the accuracy of clonal assignment as a function of genomic distinctiveness, simulating data where 5, 10, 50, 100, 500, and 1000 genes resided in regions with different copy number between clones. We discovered that with as few as 10–50 genes distinguishing clones, clonealign can still accurately assign cells to clones with a median AUC >0.8 (Fig. 1d). We further found clonealign to be robust across a range of realistic scenarious, including number of clones, minor clone frequency, and RNA-seq data quality (Additional file 1: Supplementary Text Section 1). We also assessed the runtime efficiency of clonealign on a virtual machine, finding the time required to perform inference on a large dataset (10,000 cells, 800 genes, 16 clones) taking just over 40 min (Additional file 1: Supplementary text section 1).

We next investigated the capacity of our approach to reveal clone-specific phenotypic properties in real cancer data, using the serially passaged triple-negative breast cancer patient-derived xenograft SA501 as a substrate. SA501 exhibits a complex clonal architecture and reproducible clonal dynamics over successive xenograft passages [14]. Thus, it is an ideal model system to exemplify clone-specific gene expression. We previously described single-cell DLP DNA-seq for SA501X3F [1], a copy number analysis of which identified three genotypically distinct clones (A, B, and C) with prevalences 82.3%, 10.8%, and 6.9% respectively, with clone A further expanding in subsequent passages.

**Fig. 2:**
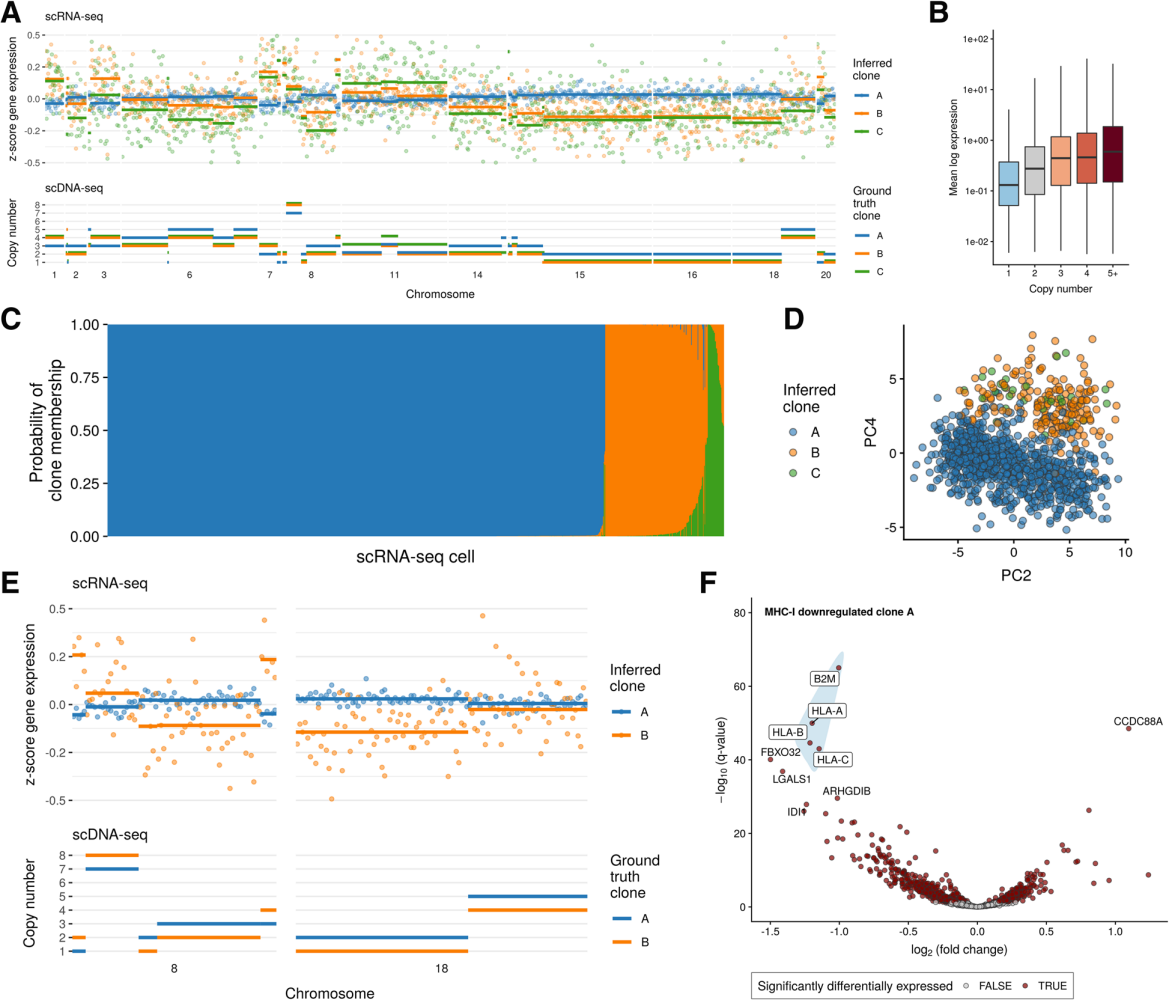
Inferring clone-dependent gene expression in SA501 triple-negative breast cancer xenograft. **a** Clone-specific copy number for ground truth clones in scDNA-seq (bottom) and clone-specific *z*-score expression for clonealign inferred clones in scRNA-seq (top) for regions exhibiting inter-clone copy number aberrations. In every copy number segment except one, when the copy number for a given clone is higher than others, then on average the normalized gene expression is also higher. **b** The mean log expression as a function of copy number across all clones. **c** Clone assignment probabilities for 1152 single-cell RNA-seq profiles across three clones. clonealign confidently assigns cells to clone A, with some cells exhibiting high assignment uncertainty between clones B and C. **d** A PCA projection using only genes residing in copy number regions shows the cells clustering by clone along components 2 and 4. **e***z*-score normalized gene expression and copy number profiles for held-out data on chromosomes 8 and 18 as a function of genomic position (gene index along chromosome). In all but one copy number segment, when the copy number profile of a clone is higher, the normalized gene expression in that chromosome is also higher on average. **f** Differential expression analysis for genes residing in regions whose copy number is identical between clones highlights downregulation of MHC class I proteins

We linked gene expression to clones in SA501 by generating single-cell RNA-seq from the SA501X2B xenograft passage using the 10X genomics chromium platform and assigned each cell to a clone (A, B, or C) using clonealign. One thousand one hundred fifty-two single cells post-QC (see the “Methods” section) were assigned to clones A, B, and C with prevalence of 80.7%, 16.7%, and 2.6%, closely matching the expected proportions inferred from the single-cell DNA-seq (82.3%, 10.8%, and 6.9%). A genome-wide view of the clone-specific copy number and expression profiles reveals a strong dosage effect as modeled by clonealign in all but one region (Fig. 2a, b). The clone assignments are highly confident for clone A but some cells exhibit uncertainty of assignment between clones B and C (Fig. 2c), reflecting a combination of having more cells in clone A as well as more similar expression profiles of B and C but distinct expression profiles of (B or C) relative to A. This latter explanation is further supported in a PCA projection using only genes residing in chromosome regions with variable copy number between clones (Fig. 2d).

We next sought to validate the clonealign assignments by both testing the internal consistency of our model and with a held out, orthogonal data source. We re-inferred the clones for SA501X2B using genes from all chromosomes except 8 and 18. If both the clone assignments and the expression-CNA assumption are correct, then the expression of genes on the held-out chromosomes (8 and 18) should closely correlate with the copy number profiles of those chromosomes. In all-but-one copy number segments of the held-out chromosomes, congruency between copy number levels and normalized gene expression was observed: where the copy number profile of a clone was higher, the normalized gene expression in that chromosome was also higher and vice-versa (Fig. 2e). We formulated this into a statistical test asking if given the clone assignments and copy number profiles we can predict the expression of genes on the held-out chromosomes better than can be expected at random, with a null distribution established over permuted clone assignments. Comparing clonealign clone assignments to the null distribution with RMSE of predictions showed significantly better predictive accuracy than could be expected at random (*p*<10^−3^, Additional file 2: Figure S1). We then added a further validation measure using a loss-of-heterozygosity (LOH) analysis (see the “Methods” section) to discover if clone-specific LOH events observed in DNA space were also observed in RNA space. A single allele resulting from a genomic LOH event can only yield mono-allelically expressed transcripts [15]. Although the allele frequency data were sparse and low coverage at germline heterozygous sites, we observed an LOH event on chromosome 18 in clone B which was mono-allelically expressed in the scRNA-seq (Additional file 2: Figure S2). Finally, we quantified the robustness of clonealign to input gene selection by incrementally reducing the number of input genes both randomly and in order of variability, finding close agreement with the assignments using all genes (see the “Methods” section and Additional file 2: Figures S3 and S4).

Having established the validity of the clone assignments, we next sought to determine clone-specific phenotypes using gene expression as a proxy. We performed a differential expression analysis comparing cells assigned to clone A to those assigned to clones B and C using Limma voom [16] using genes with greater than 500 total counts in the dataset. Fifty-two percent of genes (314/594) residing in clone-specific copy numbers (CSCN) regions were differentially expressed compared to 28% of genes in regions with identical copy number (ICN) regions (1061/3757) (1905/8201). Clone A is distinguished by loss of an entire X-chromosome, but it was previously unknown whether the loss constituted the active or inactive copy. We observed downregulation of X-inactive specific transcript *XIST* (Additional file 2: Figure S5)—expressed only on the inactive X chromosome—in clone A, implying the retained chromosome is the active copy.

We next examined the differential expression of genes residing in regions with identical copy number between clones. By construction, these genes would not be impacted by gene dosage *in cis*, but may be altered through signaling networks *in trans* where upstream transcriptional regulators lie in copy number altered regions. We found systematic downregulation of the MHC class I cell surface proteins in clone A (Fig. 2f and Additional file 2: Figure S6) along with *β*_2_ microglobulin (*B2M*), suggesting a clone-specific deficiency in presenting intra-cellular proteins to cytotoxic T cells, and therefore a putative mechanism by which clone A progressively dominates the SA501 xenograft tumors in subsequent passages. Loss of MHC expression is a mechanism of tumor immune escape [17, 18], and our results indicate this may be selected for despite the immune-deficient environment of the murine host. Importantly, clone A did not exhibit LOH in any HLA gene in clone A (Additional file 2: Figure S7), implying MHC class-I downregulation is due to transcriptional pathway alterations.

We supplemented our differential expression analysis with a variance component analysis ([19] and see the “Methods” section) to partition gene expression variation into either clone-specific or intrinsic/residual. This revealed genes whose expression variation was governed by genomic state (clonality), such as CD44 antigen—a marker of tumorigenic cancer cells [20]—of which around a quarter of expression variation is clone-specific (Additional file 2: Figure S8). To elucidate which pathways show clone-dependent regulation, we performed a gene set enrichment analysis [21] on all genes ranked by proportion of regulation explained by genomic state. Clone-specific immune response (Fig. 2f), including pathways involved in MHC class I-mediated antigen presentation were highly ranked. To discover if any transcriptional states existed within clone assignments, we performed an intra-clonal clustering of the scRNA-seq data using SC3 [22] with *k*=2 clusters and called cell cycle states using Cyclone [23]. We found clusters within each clone largely separated based on G2M score (Additional file 2: Figure S9), implying the largest source of intra-clonal variation corresponds to cell cycle stage.

**Fig. 3:**
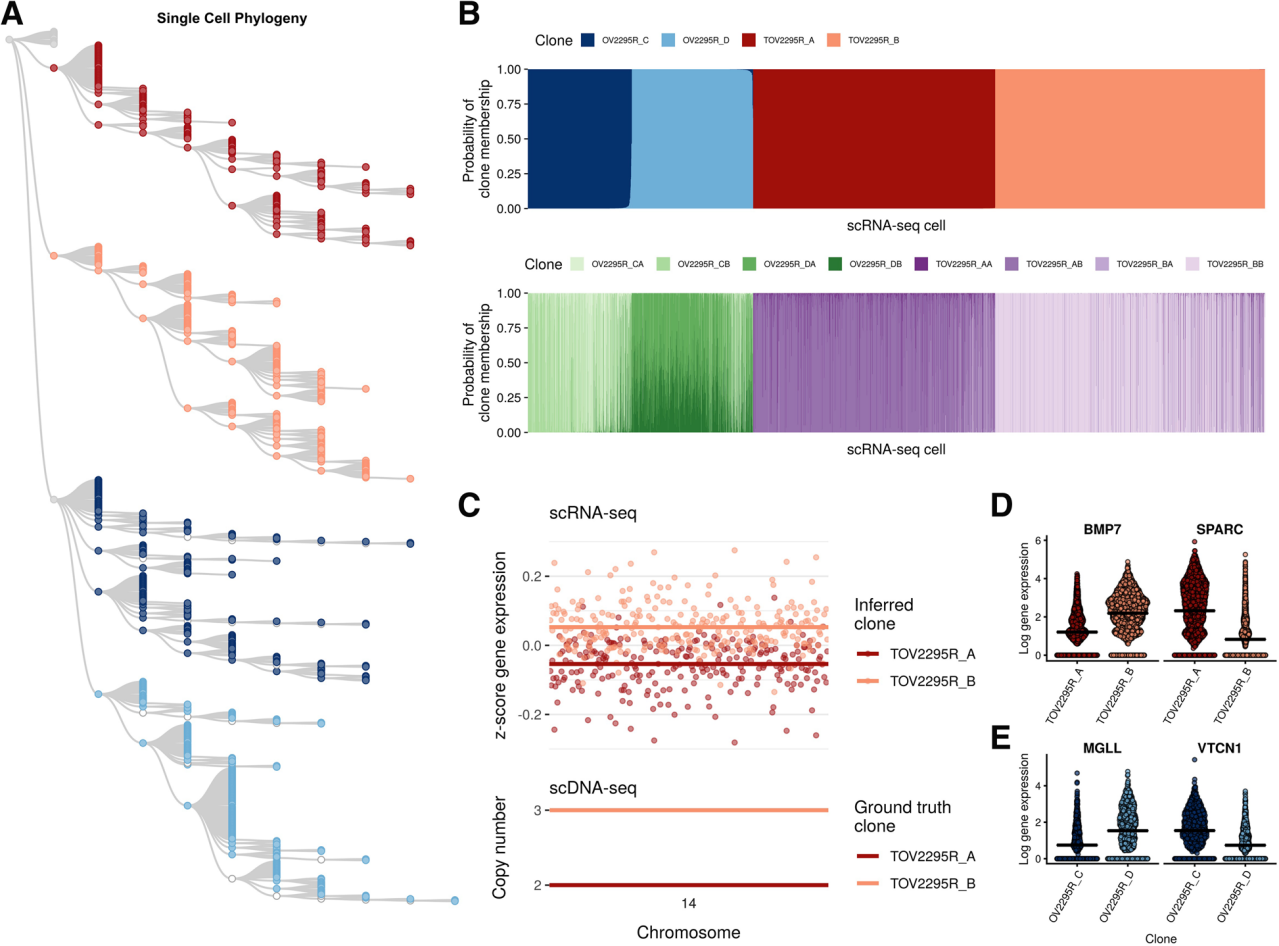
Clone-specific gene expression in high-grade serous ovarian cancer cell lines. **a** Single-cell phylogeny for the OV2295R and TOV2295R HGSC cell lines inferred using a Latent Tree Model divided into four clones (TOV2295R_A, TOV2295R_B, OV2295R_C, OV2295R_D). **b** The scRNA-seq clone assignments for the four clone model (top), then sub-divided into eight clones (bottom). **c** Expression-CNA relationship on two held out chromosomes for TOV2295R validates the clonealign fit. **d** Top differentially expressed genes between clones in TOV2295R and **e** OV2295R

We next applied clonealign to DLP scDNA-seq and 10X genomics scRNA-seq data from two clonally related high grade serous carcinoma (HGSC) cell lines, derived from both ascites (OV2295R) and solid tumor (TOV2295R) at relapse from the same patient [24]. We constructed a single-cell phylogeny on the derived copy number profiles from DLP+ using a Latent Tree Model [25], yielding four distinct clades (Fig. 3a). We assigned the cells as measured using scRNA-seq to the DLP+ clones using clonealign and found 1568 (47%) mapping to TOV2295R_A, 1748 (53%) to TOV2295R_B, 674 (46%) to OV2295R_C, and 786 (54%) to OV2295R_D (Fig. 3b, top). To ensure the clone assignments were accurate, we tested whether predicted clone-specific expression of genes on held out chromosome segments correlated well with the copy number profiles of those genes (Fig. 3c and Additional file 2: Figures S10-S12), and found these assignments to be robust to the choice of input gene (Additional file 2: Figures S13-S16). Differential expression analysis in TOV2295R identified 947/1523 (62%) genes in CSCN regions and 2362/5802 (40%) ICN regions as differentially expressed (Fig. 3d and Additional file 2: Figure S18), while in OV2295R, 307/500 (61%) and 1190/4954 (24%) were identified in CSCN and ICN regions, respectively (Fig. 3e and Additional file 2: Figure S17).

We next examined the ability of clonealign to resolve mappings as a function of phylogenetic distance between clones. In this analysis, higher levels of uncertainty in mappings between closely related clones are expected, assuming more closely related cells harbor more similar expression programs. Genomically defining a clone ultimately depends on clade-level groupings of cells that are approximately similar as a function of phylogenetic distance. We assembled a second set of clones from the OV2295R-TOV2295R phylogeny by sub-dividing each of the initial 4 clones into two (Additional file 2: Figure S19) and re-assigning each scRNA-seq cell to one of the 8 clones (Fig. 3b, bottom). We then computed Euclidean distance of each clone to its nearest neighbor and clone assignment probability for each cell. We found—as expected—a strong anti-correlation between the similarity of clones in genome space and the certainty with which cells are assigned to them (Additional file 2: Figure S20), demonstrating the analytical challenges of segregating cells into highly similar clones based on gene expression data alone. We further repeated the intra-clonal clustering analysis (as above for SA501), clustering each clone into two distinct groups separately and computing cell cycle phases. As with SA501, we found that in three of the four clones resultant clusters corresponded to cell cycle phase (Additional file 2: Figures S21 and S22), implying the largest genome-independent source of expression variation corresponds to cell cycle stage.

## Discussion and conclusions

Our results establish a scalable statistical framework for assigning cells measured using scRNA-seq to cancer clones measured independently using shallow scDNA-seq. We expect this approach can be used ubiquitously in the field of single-cell biology including extensions for other multi-modal approaches such as methylation-transcription and chromatin accessibility-transcription.

However, there are certain situations in which clonealign cannot be applied. While it is estimated that 60–80% of cancers exhibit the complex structural genomic rearrangements required to apply clonealign [26, 27], some cancers have quiescent genomes and are devoid of copy number changes. For example, cancers such as karyotypically normal AML, sarcomas, and other pediatric malignancies without genomic instability would not generate the genomic/transcriptomic signals modeled by clonealign [28].

Furthermore, the focus of this work has been on linking transcriptional measurements to genomically defined clones assuming only a copy-number dosage effect on transcript abundance. While the clonealign model allows for integration of allelic imbalance information caused by clone-specific LOH events, the sparse expression of germline heterozygous variants detected by the 10X chromium 3 ^′^ assay demonstrated here makes such information uninformative (Additional file 2: Supplementary text section 3). However, full-transcript-length single-cell RNA sequencing technologies such as Smart-seq2 [29] would allow for further refinement of clonal assignment and represent the appropriate use-case of clonealign’s incorporation of allelic imbalance information.

However, the concepts introduced in the clonealign model provide a basis for future studies of the integration of genomic data from independently sampled assays. At the edge of the field, sparse in situ measurements of transcription integrated with independent disaggregated sampling of single-cell genomes are providing a route to studying spatial context of co-located cell populations [30]. Finally, there is an emergence of commercial platforms whereby single-cell, kit-based assays for methylation, transcription, and genome copy number are becoming widely available to the research community. In all of these settings, clonealign and future derivatives will provide a statistical framework to help interpret the cellular constituents of cancer, their fitness, and their phenotypes.

## Methods

### Clonealign: model formulation and inference

We begin with an *N*×*G* matrix of expression raw read counts ***Y*** for *N* cells and *G* genes, and a *G*×*C* matrix ***Λ***=(*λ*_*gc*_) of clone specific copy numbers for *C* clones and *G* genes. Such a copy number matrix is typically obtained by phylogenetic analysis of single-cell CNV data, followed by cutting of the phylogenetic tree to produce *C* clones or clades. The goal of inference is to assign each of the *N* cells as measured in RNA-space to one of the *C* clones as measured in DNA-space.

For each cell *n*=1,…,*N*, we introduce a categorical assignment variable *z*_*n*_ defined such that

1 $$ z_{n} = c\, \text{ if cell}\ n \text{ is assigned to clone } c  $$

for *c*=1,…,*C*. Our assumption is that *y*_*ng*_—the expression of gene *g* in cell *n*—will be dependent on the copy number of the gene in the clone to which *n* is assigned, i.e., $\mathbb {E} \propto \mu _{g} f(\lambda _{gc})$ where *μ*_*g*_ is the per-copy expression of gene *g* and *f* is a *dosage function* that maps the copy number of a gene to a multiplicative factor of expression. While this function is a priori unknown and joint estimation with clonal populations would lead to an unidentifiable model, we can encode some basic assumptions about gene dosage into our specification of *f*. We assume that if the copy number change is small, it will lead to a proportional change in expression, e.g., a copy number of 3 could conceivably lead to $\frac {3}{2}\times $ more expression. Conversely, we assume that if the copy number change is large, e.g., if a clone has copy number 12 in a particular region, the cells will have a compensatory mechanism such that fewer than $\frac {12}{2} \times $ transcripts are produced, and that this is capped at an upper limit. With these considerations in mind, we specify *f* as $ f(\lambda) = \left \{\begin {array}{ll}\lambda & \text {if } \lambda < \zeta \\ \zeta & \text {if } \lambda \geq \zeta, \end {array}\right.$where in our analyses we fix *ζ*=6. We leave as future work more sophisticated approaches such as inferring *f* from joint genomic-transcriptomic assays or marginalizing out *ζ* in Bayesian models.

We next specify the exact likelihood model for clonealign. There is a subtlety in modeling RNA-seq data as outlined in [31] in that the expression of each gene is measured relative to all other genes in a given library multiplied by the sequencing depth of that library. Taking this into account is of critical importance to our problem as if a highly expressed gene sits in a high copy number region in a clone it will cause a *decrease* in expression of all other genes. Therefore, the expected count of gene *g* in cell *n* conditional on that cell being assigned to clone *c* is given by:

2 $$ \begin{aligned} &\mathbb{E}[y_{ng}|z_{n}=c] =\\ &\underbrace{s_{n}}_{\substack{\text{Cell read} \\ \text{depth}}} \frac{\overbrace{\mu_{g}}^{\substack{\text{Per-copy} \\ \text{expression}}} \times \text{ \;} f(\overbrace{\lambda_{gc}}^{\substack{\text{Copy} \\ \text{number}}}) \times e^{\overbrace{\boldsymbol{x}_{n} \cdot \boldsymbol{\beta}_{g}^{T}}^{\substack{\text{Known} \\ \text{covariates}}} + \overbrace{\boldsymbol{\psi}_{n} \cdot \boldsymbol{w}_{g}^{T}}^{\substack{\text{Residual} \\ \text{expression}}}}} {\underbrace{\sum\limits_{g'=1}^{G} \mu_{g'} f(\lambda_{g'c}) e^{\boldsymbol{x}_{n} \cdot \boldsymbol{\beta}_{g'}^{T} + \boldsymbol{\psi}_{n} \cdot \boldsymbol{w}_{g'}^{T}}}_{\text{Total RNA normalization}}} \end{aligned}  $$

where *s*_*n*_ is the total read depth size of cell *n*.

The inner product $\boldsymbol {\psi }_{n} \cdot \boldsymbol {w}_{g}^{T}$ between the row vectors ***ψ***_*n*_ of a *N*×*Q* matrix ***Ψ*** and the row vectors ***w***_*g*_ of a *G*×*Q* matrix ***W*** introduces structured noise to the model and avoids forcing all expression variation to be explained in terms of copy number variation, which is untrue in practice. This term is analogous to representing observed data as the product of two low-rank matrices in models such as factor analysis and linear mixed-effects models. By default, we set *Q*=6 though if fewer than 100 genes are used as input we set *Q*=1 to avoid “over-explaining” the expression variance with residual factors. We ensure the model is weakly identifiable by imposing priors $\boldsymbol {\psi }_{n} \sim \mathcal {N}(0,1) \; \forall n$ and factor-specific priors $w_{gk} \sim \mathcal {N}\left (0, \chi _{k}^{-1}\right), \chi _{k} \sim \text {Gamma}(2,1)$.

clonealign also allows for the incorporation of known covariates encoded in the *N*×*P* matrix ***X*** with unknown *G*×*P* coefficients matrix ***B***, with the inner product $\boldsymbol {x}_{n} \cdot \boldsymbol {\beta }_{g}^{T}$ affecting the mean in a similar way to the random effects as above. The covariates ***x***_*n*_ can encode known groupings of cells such as experimental batch effects, or additional biological information such as cell cycle stage that can either be inferred experimentally or from the gene expression data using methods such as scran [32].

We impose a negative binomial likelihood as is commonly used to model both RNA-seq [31, 33] and single-cell RNA-seq data [34] with a mean given by Eq. (2). We model the dispersion parameter *ϕ* as a non-parametric function of the mean parameter using radial basis function (RBF) kernels as proposed in a recent work [35]. Specifically, we set:

3 $$ \phi(\mu) = \sum\limits_{i=1}^{M} a_{i} \exp(-b(\mu - c_{i})^{2})  $$

where the location *c*_*i*_ and with *b* of each basis function is a fixed hyperparameter and the amplitude of each *a*_*i*_ is jointly inferred from the data. We fix *M*=20 by default and evenly space *c*_*i*_ from the minimum to maximum raw count values and set *b*=1/(2*δ*^2^) where *δ* is the distance between consecutive bases.

The model as defined in 2 is invariant to rescalings of all *μ*, so we fix *μ*_1_=1 and the interpretation of the remaining *μ*_2_,…,*μ*_*G*_ is the per-copy expression relative to gene 1 with a prior $\log \mu _{g} \sim \mathcal {N}(0,1)$. The total read depth *s*_*n*_ can either be jointly inferred with the model or fixed beforehand.

Inference is performed using mean field variational Bayes (see, e.g., [36]). Briefly, given the joint distribution *p*(***x***,***θ***) of the data ***x*** and model parameters ***θ***, we seek to find a variational distribution *q*(***θ***|***ζ***) where ***ζ*** are the variational parameters that approximates the posterior *p*(***θ***|***x***) by minimizing KL[*q*(***θ***|***ζ***)||*p*(***θ***|***x***)], the Kullbach-Leibler divergence between the variational and posterior distributions, which is equivalent to minimizing the evidence lower bound (ELBO). The non-conjugate nature of the model in Eq. 2 requires us to compute a Monte Carlo estimate of the KL divergence that we can optimize by computing low-variance gradients using the reparametrization trick [37].

Specifically, we posit an approximating distribution of the form $q(\boldsymbol {z}, \boldsymbol {\mu }) = \prod _{n} q(z_{n}) \prod _{g} q(\mu _{g})$ for the clone assignment and mean expression variables respectively and optimize all other model parameters as variational parameters in a similar manner to [37]. The approximating distribution for the clone assignments is categorical of the form *q*(*z*_*n*_=*c*)=*φ*_*nc*_. The approximating distribution for the mean expression parameters is given by a continuously differentiable invertible transform of standard Gaussian noise $\epsilon \sim \mathcal {N}(0,1)$ by *μ*_*g*_= exp(*ν*_*g*_+*ρ*_*g*_*ε*). While the expectation over *q*(***z***) can be taken analytically, to calculate the expectation with respect to *q*(***μ***), we must compute a Monte Carlo estimate by drawing *S* samples ***μ***^(*s*)^∼*q*(***μ***), where we set *S*=1 following previous literature [37].

Optimization is performed using the Adam optimizer [38] as implemented in Tensorflow. Convergence is assessed by monitoring the ELBO with the model converged when the change between consecutive iterations drops below 10^−6^*%*. clonealign is open source and available online at http://www.github.com/kieranrcampbell/clonealign.

### Incorporating alleleic imbalance information

We can leverage allelic imbalance information in scRNA-seq data to further refine clonotype assignment. For expressed heterozygous germline SNPs in regions of clone-specific copy number, if there is a clone-specific LOH event, then the allelic ratios will be biased towards 0 (loss of alt) or 1 (loss of ref) compared to diploid regions where the allelic ratio should be centered around $\frac {1}{2}$. Note that we assume (i) the scDNA-seq is too shallow to phase variants, and (ii) there is no copy-neutral LOH. If the user believes assumption (ii) is violated by inspecting the scDNA-seq reads, then clonealign should be run using gene expression data alone.

We define the augmented statistical model as follows: let *a*_*nv*_ and *r*_*nv*_ be the alt and ref counts for (germline heterozygous) variant *v* in cell *c* for *n*=1,…,*N* and *v*=1,…,*V*. Further, let *λ*_*vc*_ be the copy number at variant *v* in clone *c* inferred from the scDNA-seq data. Then the likelihood conditioned on the clone is given by:

4 $$ p(a_{nv}, r_{nv}) = \left\{ \begin{array}{ll} D_{\text{LOH}}(a_{nv}, r_{nv}) & \text{if} \lambda_{vc} = 1 \\ D_{\text{HET}}(a_{nv}, r_{nv}) & \text{if} \lambda_{vc} = 2 \end{array}\right.  $$

where

5 $$ \begin{aligned} D_{\text{HET}}(a_{nv}, r_{nv}) & = \text{BetaBinomial}(a_{nv}, a_{nv} + r_{nv} | \alpha = 2, \beta = 2) \\ D_{\text{LOH}}(a_{nv}, r_{nv}) & = \frac{1}{2}\text{BetaBinomial}(a_{nv}, a_{nv} + r_{nv}|\alpha = 0.1,\beta = 1.9) \\ & + \frac{1}{2} \text{BetaBinomial}(a_{nv}, a_{nv} + r_{nv} | \alpha = 1.9, \beta = 0.1) \end{aligned}  $$

The use of the beta binomial model is motivated by the observation that the read counts will follow a binomial distribution but the exact number of successes (alt read fraction) is not known exactly due to sequencing errors and RNA editing, so we marginalize over this to get the given the observation model. The distribution *D*_HET_ places mass around an alternate allele fraction of $\frac {1}{2}$ while *D*_LOH_ places its mass at 0 and 1. The variance calibration leading to the exact choice of parameters is taken from a recent study of clone-specific allele expression in scRNAseq [39]. The likelihood induced by Eq. 5 is then multiplied iid and added to the log joint probability of the data and parameters for variational inference, when SNV data is available. A dockerized workflow to produce the required variant by clone and variant by cell matrices from the output of the 10X CellRanger software and HMMCopy [15] respectively is available at http://www.github.com/kieranrcampbell/snvworkflow.

### Simulations

To ensure all simulations were as realistic as possible, the clonealign model was fitted to the SA501 dataset giving an empirical distribution of the model parameters and data *p*(***Φ***,***μ***,***Λ***)*p*(***s***). We then simulated from the clonealign model, sampling from the empirical distribution of model parameters. For clonealign, we considered five different simulation scenarios, where each scenario represents the marginal effect as the full combination of effects would be computationally infeasible. All simulations reported the area under the receiver operator curve (AUC) as a measure of accuracy, except for varying the number of clones where we use the accuracy as the metric (proportion of clones called as correct).

**Varying proportion of genes with dosage effect**

For each simulation, a certain proportion *π*=0.1,0.2,0.3,…,0.9 of genes were simulated with a CN-expression dependency, while the expression of the remaining 1−*π* proportion had an expression independent of copy number, achieved by setting the copy number to 2 for all clones during simulation of the expression, but providing the true copy number during inference as clonealign does not know a priori which genes exhibit a CN expression dependency. Datasets were simulated for two clones corresponding to the A and B clones from SA501.

**Varying how genomically distinct clones are**

The number of genes distinguishing clones in clone-specific copy number regions was varied from 2, 5, 10, 50, 100, 500, and 1000 for 1000 cells and 2 clones.

**Varying the number of clones**

The number of clones simulated was set to 2, 4, 8, 16, 32, and 64 for 200 and 800 genes and 1000 cells.

**Varying the minor clone frequency**

The minor clone frequency was varied among 1*%*,5*%*,10*%*,20*%*, and 50% for 200 and 800 genes and 1000 cells.

**Varying the quality of the scRNA-seq data**

We subsampled the simulated 10X data from the original dataset size of 0.86 reads per gene per cell down to 1*%*,5*%*,10*%*, and 50% for 200 and 800 genes and 1000 cells.

### Bioinformatics analysis

For all scRNA-seq data expression, estimates were obtained from raw read counts using CellRanger (version 2.0.1 for SA501X2B and version 2.1.0 for (T)OV2295R) aligned to hg19. Quality control of SA501X2B cells removed those with fewer than 1000 counts or 350 expressed genes in regions of distinct copy number between clones A, B, and C. Clone-specific copy number calls were created according to [1]. X-chromosome genes were removed prior to clonealign analysis as the expression-copy number assumption will be violated if the deleted/amplified X copy is inactive. For OV2295R and TOV2295R, cells were retained with total UMIs greater than 20,000, and total number of genes detected between 3000 and 7500. Copy number calls for scDNA-seq were performed using HMMCopy version 1.22.0 and a phylogeny constructed using a latent tree model. The clone-specific copy number was constructed as the median copy number of all cells in a clone at a given position. Genes on the X-chromosome were removed as before.

Differential expression (DE) analysis was performed using Limma Voom [16] version 3.36.0. For SA501X2B, genes with greater than 100 total counts were retained for DE. For both OV2295R and TOV2295R, genes with greater than 500 total counts were retained for DE as up to this threshold the mean-dispersion relationship reported by Limma Voom was visually a poor fit. All *p* values were corrected for multiple hypothesis testing using the Benjamini-Hochberg procedure.

For the SA501 LOH analysis, bulk whole-genome DNA sequencing as previously described in [14] was aligned to hg19 using BWA aln version 0.7.10 after which germline LOH alleles were identified using samtools 1.7 mpileup followed by VarScan 2.3.9 [40] mpileup2snp command (default settings). Single-cell RNA and DNA-seq profiles were merged into pseudobulk clones using samtools version 1.7 and reads mapping to ref and alt alleles at positions identified as germline heterozygous called using Varscan mpileup2cns command with default settings other than setting –min-avg-qual 5 on the merged scRNA-seq to increase the number of callable positions. Regions in the pseudobulk pileups were called LOH using Titan version 1.16.0 [41]. We compared the major allele frequency in the region of chromosome 18 from position 5.5×10^7^ onwards, finding a significantly reduced major allele frequency in clone A in both DNA (*p*=3.7×10^−51^) and in RNA (*p*=5.9×10^−4^), both using one-sided Wilcoxon rank-sum test.

The results of the simulations in Fig. 1d suggest that the higher the latent proportion of genes that exhibit CN-gene dependency, the more accurate our inference. While the set of genes that exhibit such dependency is unknown a priori and most likely cancer and even patient specific, it is possible to select a set of genes that are more likely to exhibit such interactions based on previous studies. For example, we took the copy number and expression data from both the BRCA and OV cohorts from The Cancer Genome Atlas (TCGA, [42]) and regressed log-expression on log*R* (relative copy number). We found the vast majority of genes exhibited a positive correlation with log*R* (Additional file 2: Figures S21 and S22). It is possible to use only these genes in analyses such as clonealign.

To test the robustness of clonealign to input gene selection for the SA501, TOV2295R, and OV2295R datasets, we re-fitted clonealign excluding the bottom *p*% of least variable genes (as defined in log-expression space), for *p*∈{10,20,40,60,80,90}, and compared the concordance in clone assignments between fits. The results can be seen as *alluvial* plots in Additional file 2: Figures S4, S12 and S13, demonstrating that clonealign is highly robust to the input gene selection and that in general up to 60% of the least variable genes may be removed before the clone assignments begin to significantly change.

We further assessed the stability of clonealign clone assignments to random removal of genes for the SA501, OV2295, and TOV2295 datasets. For each, we removed a proportion (0.3, 0.5, 0.7, 0.9) of genes at random across 10 replicates and computed the precision and recall as if the fits using all genes represented the true clonal assignments. While the results exhibit decreasing agreement with increasing number of genes removed and variability across datasets, in general, up to 30% of genes could be removed to maintain average precision and recall >0.8 for all clones (Additional file 2: Figures S4, S15, S16).

To rank genes by proportion of variance explained by clonality in SA501, the full dataset was subsetted to remove any ribosomal genes and those on the X chromosome (due to entire chromosome loss). We further only considered genes whose variance in log-expression was greater than the mean variance over all genes to avoid spurious associations (i.e., if a gene is expressed only in a single-cell, its entire expression variation is trivially explained by clonality). The proportion of expression variation was calculated using the aov function in R. Gene Set Enrichment Analysis was then performed using the fgsea package [43] using all ReactomeDB pathways with genes ranked according to proportion of expression variance explained by clonality.

### Cell lines and tissue preparation

OV2295 and TOV2295 cells were cultured in Dulbecco’s modified Eagle’s medium supplemented with 10% FBS. Patient-derived xenografts were generated under the tumor tissue repository (TTR-H06-00289) protocol, which fulfills the requirements of UBC BCCA Research Ethics Board. All animal studies were approved by the Animal Care Committee at the University of British Columbia. Xenografts were transplanted subcutaneously into female NOD/SCID interleukin-2 receptor gamma null (NSG) and NOD Rag-1 null interleuki–2 receptor gamma null (NRG) mice as previously described (Eirew et al., 2015). Harvested tumors were viably frozen in DMEM containing 45% FBS and 6% DMSO.

**Single-cell RNA sequencing** Thawed samples were digested for 2 h with collagenase/hyaluronidase, and single cells were FACS sorted for viability by propidium iodide negativity. Single-cell suspensions were loaded onto the 10X genomics single-cell controller and libraries prepped according to the Chromium Single Cell 3”’ Reagent v2 Chemistry kit standard protocol. Libraries were then sequenced on an Illumina Nextseq500/550 with 42 bp paired end reads. Cell Ranger 2.0 was used to perform demultiplexing, alignment, and counting.

**Single-cell DNA sequencing** Single-cell suspensions were stained with LIVE/DEAD Fixable Red Dead Cell Stains (ThermoFisher) and using a cellenONE (Cellenion), single cells dispensed into each well on a nanowell chip containing two unique dual indices [11]. Libraries were generated using a one-pot transposase chemistry (Nextera DNA Library Preparation Kit, Illumina) as previously described [1, 11]. Briefly, spotted cells were lysed overnight, followed by tagmentation, neutralization, and a sample index PCR.

## Additional files

Additional file 1 Supplementary text. (PDF 108 kb)

Additional file 2 Supplementary figures. (PDF 6381 kb)

## References

[1] Zahn H, Steif A, Laks E, Eirew P, VanInsberghe M, Shah SP, Aparicio S, Hansen CL (2017). Scalable whole-genome single-cell library preparation without preamplification. Nat Methods.

[2] Zheng GXY, Terry JM, Belgrader P, Ryvkin P, Bent ZW, Wilson R, Ziraldo SB, Wheeler TD, McDermott GP, Zhu J, Gregory MT, Shuga J, Montesclaros L, Underwood JG, Masquelier DA, Nishimura SY, Schnall-Levin M, Wyatt PW, Hindson CM, Bharadwaj R, Wong A, Ness KD, Beppu LW, Deeg HJ, McFarland C, Loeb KR, Valente WJ, Ericson NG, Stevens EA, Radich JP, Mikkelsen TS, Hindson BJ, Bielas JH (2017). Massively parallel digital transcriptional profiling of single cells. Nat Commun.

[3] Jahn K, Kuipers J, Beerenwinkel N (2016). Tree inference for single-cell data. Genome Biol.

[4] Smith MA, Nielsen CB, Chan FC, McPherson A, Roth A, Farahani H, Machev D, Steif A, Shah SP (2017). E-scape: interactive visualization of single-cell phylogenetics and cancer evolution. Nat Methods.

[5] Schelker M, Feau S, Du J, Ranu N, Klipp E, MacBeath G, Schoeberl B, Raue A (2017). Estimation of immune cell content in tumour tissue using single-cell RNA-seq data. Nat Commun.

[6] Tellez-Gabriel M, Ory B, Lamoureux F, Heymann M-F, Heymann D (2016). Tumour heterogeneity: the key advantages of single-cell analysis. Int J Mol Sci.

[7] Mitra AK, Stessman H, Linden MA, Van Ness B (2014). Single-cell transcriptomics identifies intra-tumor heterogeneity in human myeloma cell lines. Blood.

[8] Shaffer SM, Dunagin MC, Torborg SR, Torre EA, Emert B, Krepler C, Beqiri M, Sproesser K, Brafford PA, Xiao M (2017). Rare cell variability and drug-induced reprogramming as a mode of cancer drug resistance. Nature.

[9] Macaulay IC, Haerty W, Kumar P, Li YI, Hu TX, Teng MJ, Goolam M, Saurat N, Coupland P, Shirley LM (2015). G&t-seq: parallel sequencing of single-cell genomes and transcriptomes. Nat Methods.

[10] Dey SS, Kester L, Spanjaard B, Bienko M, Van Oudenaarden A (2015). Integrated genome and transcriptome sequencing of the same cell. Nat Biotechnol.

[11] Laks E, Zahn H, Lai D, McPherson A, Steif A, Brimhall J, Biele J, Wang B, Masud T, Grewal D, et al.Resource: Scalable whole genome sequencing of 40,000 single cells identifies stochastic aneuploidies, genome replication states and clonal repertoires. bioRxiv. 2018:411058.

[12] Curtis C, Shah SP, Chin S-F, Turashvili G, Rueda OM, Dunning MJ, Speed D, Lynch AG, Samarajiwa S, Yuan Y (2012). The genomic and transcriptomic architecture of 2,000 breast tumours reveals novel subgroups. Nature.

[13] Han KY, Kim K-T, Joung J-G, Son D-S, Kim YJ, Jo A, Jeon H-J, Moon H-S, Yoo CE, Chung W (2018). Sidr: simultaneous isolation and parallel sequencing of genomic dna and total rna from single cells. Genome Res.

[14] Eirew P, Steif A, Khattra J, Ha G, Yap D, Farahani H, Gelmon K, Chia S, Mar C, Wan A (2015). Dynamics of genomic clones in breast cancer patient xenografts at single-cell resolution. Nature.

[15] Ha G, Roth A, Lai D, Bashashati A, Ding J, Goya R, Giuliany R, Rosner J, Oloumi A, Shumansky K (2012). Integrative analysis of genome-wide loss of heterozygosity and monoallelic expression at nucleotide resolution reveals disrupted pathways in triple-negative breast cancer. Genome Res.

[16] Law CW, Chen Y, Shi W, Smyth GK (2014). voom: Precision weights unlock linear model analysis tools for rna-seq read counts. Genome Biol.

[17] Garrido F, Aptsiauri N, Doorduijn EM, Lora AMG, van Hall T (2016). The urgent need to recover MHC class i in cancers for effective immunotherapy. Curr Opin Immunol.

[18] Garrido C, Paco L, Romero I, Berruguilla E, Stefansky J, Collado A, Algarra I, Garrido F, Garcia-Lora AM (2012). MHC class i molecules act as tumor suppressor genes regulating the cell cycle gene expression, invasion and intrinsic tumorigenicity of melanoma cells. Carcinogenesis.

[19] Arnol D, Schapiro D, Bodenmiller B, Saez-Rodriguez J, Stegle O. Modelling cell-cell interactions from spatial molecular data with spatial variance component analysis. bioRxiv. 2018;:265256. 10.1101/265256. 10.1016/j.celrep.2019.08.077 PMC6899515 31577949

[20] Al-Hajj M, Wicha MS, Benito-Hernandez A, Morrison SJ, Clarke MF (2003). Prospective identification of tumorigenic breast cancer cells. Proc Natl Acad Sci.

[21] Subramanian A, Tamayo P, Mootha VK, Mukherjee S, Ebert BL, Gillette MA, Paulovich A, Pomeroy SL, Golub TR, Lander ES (2005). Gene set enrichment analysis: a knowledge-based approach for interpreting genome-wide expression profiles. Proc Natl Acad Sci.

[22] Kiselev VY, Kirschner K, Schaub MT, Andrews T, Yiu A, Chandra T, Natarajan KN, Reik W, Barahona M, Green AR (2017). Sc3: consensus clustering of single-cell rna-seq data. Nat Methods.

[23] Scialdone A, Natarajan KN, Saraiva LR, Proserpio V, Teichmann SA, Stegle O, Marioni JC, Buettner F (2015). Computational assignment of cell-cycle stage from single-cell transcriptome data. Methods.

[24] Létourneau IJ, Quinn MC, Wang L-L, Portelance L, Caceres KY, Cyr L, Delvoye N, Meunier L, de Ladurantaye M, Shen Z (2012). Derivation and characterization of matched cell lines from primary and recurrent serous ovarian cancer. BMC Cancer.

[25] Farahani H. Latent tree model. 2018. http://www.github.com/shahcompbio/LTM. Accessed 17 May 2018.

[26] Bakhoum SF, Cantley LC (2018). The multifaceted role of chromosomal instability in cancer and its microenvironment. Cell.

[27] Beroukhim R, Mermel CH, Porter D, Wei G, Raychaudhuri S, Donovan J, Barretina J, Boehm JS, Dobson J, Urashima M (2010). The landscape of somatic copy-number alteration across human cancers. Nature.

[28] Kawankar N, Korgaonkar S, Kerketta L, Madkaikar M, Jijina F, Ghosh K, Vundinti BR (2012). Dna copy number changes and immunophenotype pattern in karyotypically normal acute myeloid leukemia patients from an indian population. Genet Test Mol Biomark.

[29] Picelli S, Faridani OR, Björklund ÅK, Winberg G, Sagasser S, Sandberg R (2014). Full-length RNA-seq from single cells using smart-seq2. Nat Protoc.

[30] Achim K, Pettit J-B, Saraiva LR, Gavriouchkina D, Larsson T, Arendt D, Marioni JC (2015). High-throughput spatial mapping of single-cell RNA-seq data to tissue of origin. Nat Biotechnol.

[31] Robinson MD, Oshlack A (2010). A scaling normalization method for differential expression analysis of RNA-seq data. Genome Biol.

[32] Lun AT, McCarthy DJ, Marioni JC. A step-by-step workflow for low-level analysis of single-cell RNA-seq data with bioconductor. F1000Research. 2016;5. 10.12688/f1000research.9501.1 PMC5112579 27909575

[33] Love MI, Huber W, Anders S (2014). Moderated estimation of fold change and dispersion for RNA-seq data with DESeq2. Genome Biol.

[34] Risso D, Perraudeau F, Gribkova S, Dudoit S, Vert JP (2018). A general and flexible method for signal extraction from single-cell RNA-seq data. Nat Commun.

[35] Eling N, Richard AC, Richardson S, Marioni JC, Vallejos CA (2018). Correcting the mean-variance dependency for differential variability testing using single-cell rna sequencing data. Cell Syst.

[36] Blei DM, Kucukelbir A, McAuliffe JD (2017). Variational inference: a review for statisticians. J Am Stat Assoc.

[37] Kingma DP, Welling M. Auto-encoding variational bayes. arXiv preprint arXiv:1312.6114. 2013.

[38] Kingma DP, Ba J. Adam: A method for stochastic optimization. arXiv preprint arXiv:1412.6980. 2014.

[39] McCarthy DJ, Rostom R, Huang Y, Kunz DJ, Danecek P, Bonder MJ, Hagai T, Wang W, Gaffney DJ, Simons BD, et al.Cardelino: Integrating whole exomes and single-cell transcriptomes to reveal phenotypic impact of somatic variants. bioRxiv. 2018;:413047. 10.1101/413047.

[40] Koboldt DC, Zhang Q, Larson DE, Shen D, McLellan MD, Lin L, Miller CA, Mardis ER, Ding L, Wilson RK (2012). Varscan 2: somatic mutation and copy number alteration discovery in cancer by exome sequencing. Genome Res.

[41] Ha G, Roth A, Khattra J, Ho J, Yap D, Prentice LM, Melnyk N, McPherson A, Bashashati A, Laks E (2014). Titan: inference of copy number architectures in clonal cell populations from tumor whole-genome sequence data. Genome Res.

[42] Weinstein JN, Collisson EA, Mills GB, Shaw KRM, Ozenberger BA, Ellrott K, Shmulevich I, Sander C, Stuart JM, Network CGAR (2013). The cancer genome atlas pan-cancer analysis project. Nat Genet.

[43] Sergushichev A. An algorithm for fast preranked gene set enrichment analysis using cumulative statistic calculation. bioRxiv. 2016;060012. 10.1101/060012.

[44] Campbell KR, Steif A, Laks E, Zahn H, Lai D, McPherson A, Farahani H, Kabeer F, O’Flanagan C, Biele J, Brimhall J, Wang B, Walters P, Consortium I, Bouchard-Côté A, Aparicio S, Shah SP. clonealign: paper-analysis-version. 2019. https://zenodo.org/record/1892336. 10.5281/zenodo.1892336.

[45] Campbell KR, Steif A, Laks E, Zahn H, Lai D, McPherson A, Farahani H, Kabeer F, O’Flanagan C, Biele J, Brimhall J, Wang B, Walters P, Consortium I, Bouchard-Côté A, Aparicio S, Shah SP. snvworkflow: paper-analysis-version. 2019. https://zenodo.org/record/1974562. 10.5281/zenodo.1974562. Accessed 8 Oct 2018.

[46] Campbell KR, Steif A, Laks E, Zahn H, Lai D, McPherson A, Farahani H, Kabeer F, O’Flanagan C, Biele J, Brimhall J, Wang B, Walters P, Consortium I, Bouchard-Côté A, Aparicio S, Shah SP. Latent tree model software used in clonealign paper. 2019. https://zenodo.org/record/2546904. 10.5281/zenodo.2546904. Accessed 17 May 2018.

[47] Campbell KR, Steif A, Laks E, Zahn H, Lai D, McPherson A, Farahani H, Kabeer F, O’Flanagan C, Biele J, Brimhall J, Wang B, Walters P, Consortium I, Bouchard-Côté A, Aparicio S, Shah SP. 10X genomics chromium single-cell RNA-sequencing of (i) patient derived triple negative breast cancer xenograft (ii) primary tumour and ascites ovarian cancer cell lines at tumour recurrence. 2019. https://www.ebi.ac.uk/ega/studies/EGAS00001003387.

[48] Campbell KR, Steif A, Laks E, Zahn H, Lai D, McPherson A, Farahani H, Kabeer F, O’Flanagan C, Biele J, Brimhall J, Wang B, Walters P, Consortium I, Bouchard-Côté A, Aparicio S, Shah SP. Direct library preparation+ single-cell DNA-sequencing of (i) patient derived triple negative breast cancer xenograft (ii) primary tumour and ascites ovarian cancer cell lines at tumour recurrence. 2019. https://www.ebi.ac.uk/ega/studies/EGAS00001003190. Accessed 1 Nov 2017.

[49] Zahn H, Steif A, Laks E, Eirew P, VanInsberghe M, Shah SP, Aparicio S, Hansen CL. scalable whole-genome single-cell library preparation without preamplification. 2017. https://www.ebi.ac.uk/ega/studies/EGAS00001002170. Accessed 1 Nov 2017. 10.1038/nmeth.4140 28068316

[50] Campbell KR, Steif A, Laks E, Zahn H, Lai D, McPherson A, Farahani H, Kabeer F, O’Flanagan C, Biele J, Brimhall J, Wang B, Walters P, Consortium I, Bouchard-Côté A, Aparicio S, Shah SP. Processed data for clonealign: statistical integration of independent single-cell RNA and DNA-seq from human cancers. 2019. https://zenodo.org/record/2363826. 10.5281/zenodo.2363826. Accessed 17 Dec 2018.

[51] Campbell KR, Steif A, Laks E, Zahn H, Lai D, McPherson A, Farahani H, Kabeer F, O’Flanagan C, Biele J, Brimhall J, Wang B, Walters P, Consortium I, Bouchard-Côté A, Aparicio S, Shah SP. All simulated data for clonealign paper. 2019. https://zenodo.org/record/2363961. 10.5281/zenodo.2363961. Accessed 7 Dec 2018.

