# Supplementary material for: clonealign: statistical integration of independent single-cell RNA and DNA sequencing data from human cancers
Source: Genome Biol. 2019 Mar 12;20:54. doi: 10.1186/s13059-019-1645-z (PMC6417140; doi:10.1186/s13059-019-1645-z)
Supplement: Supplementary file 1 — Supplementary text. (PDF 108 kb) [file 13059_2019_1645_MOESM1_ESM.pdf]

Additional file 1: Supplementary Text for "clonealign: statistical integration of independent single-cell RNA & DNA-seq from human cancers"

January 30, 2019

1 Accuracy & scalability of clonealign through simulations

1.1 Number of clones

In this case we depart from the usual setup of simulations that consists of bootstrap sampling of the copy number data of our SA501 dataset as this did not contain enough clones for simulating greater than 3, so we simulate the copy number for each gene and clone as randomly sampled from 1,...,5. We compute the accuracy rather than AUC as there are now multiple classes. We simulated data for 1000 cells, 200 and 800 genes, and 2,4,8,16,32, and 64 clones. We see that clonaligns inferences remain highly accurate even up to 64 clones simulated (Fig S30).

1.2 Minor clone frequencies

We evaluated how robust clonealign is to the minor clone frequency for $C = 2$ clones. We simulated data for 1000 cells, 200 and 800 genes and minor clone frequencies of 0.01, 0.05, 0.1, 0.2, and 0.5. We found that clonealign exhibited high AUC across the range of minor clone frequencies (Fig S31a). However, we noticed that the accuracy (proportion of cells correctly assigned to clones) decreases with decreasing minor clone frequency (Fig S31b). Curious at the disparity, we examined the clonealign clone probabilities for cases of high AUC and low accuracy (Fig S 31c) and noted that there is still a robust decision boundary between the clones but at a miscalibrated probability - in this example all cells would be assigned to clone 2 even though all from clone 1 having higher probability than clone 2. Despite this finding, on average clonealign maintains high accuracy across the range of clonal frequencies, particularly with a larger (800) number of genes. Note that in this benchmarking we take the most likely clone even when probabilities are very close to 0.5, which may underestimate the accuracy as in practice the researcher would likely filter out cells whose assignment was uncertain.

1.3 Data quality

We randomly subsampled the simulated data to 1%, 5%, 10%, 50% of reads and recomputed the AUC using clonealign, noting that the standard simulated read depth in SA501 is 0.86 reads per cell which is close to optimal for scRNA-seq (1). Even at 5% subsample clonealign obtains a median AUC with ground truth of over 0.8, demonstrating it is robust to ultra shallow assays (Fig S32).

2 Performance

We simulated datasets for $N = 100, 200, 500, 1000, 5000, 10000$ cells, $C = 2, 4, 8, 16$ clones and $G = 200, 800$ genes, and measured the elapsed time (time from calling the `clonealign` function to clonealign completing enough iterations to achieve a change in the ELBO $< 0.0001\%$). This was performed on a "Standard D32s v3 (32 vcpus, 128 GB memory)" Microsoft Azure Virtual Machine, using a "worst case" scenario setup of a basic CPU installation - an order of magnitude speedup can be gained simply installing tensorflow using anaconda (`https://www.anaconda.com/blog/developer-blog/tensorflow-in-anaconda/`) and similar speed gains can be had from using GPUs, though as these are still somewhat niche amongst the bioinformatics community we do not include any comparisons here. We found that the computational complexity scales approximately linearly with the number of cells, clones and genes, and the upper time limit for current data sizes (10,000 cells, 800 genes, 16 clones) is around 2500 seconds or just over 40 minutes (Fig S33).

3 Incorporating allelic imbalance information in SA501 inference

We incorporated allelic imbalance information into the analysis of SA501, counting reference and alternative alleles and known heterozygous sites (methods) using the workflow at `www.github.com/kieranrcampbell/snvworkflow`. We compared the clonal assignments to those using expression information only (table S1) and found strong agreement. We then examined the extent to which cells could be assigned using allelic imbalance information only. We found that over half the cells in the sample had no usable allelic imbalance information (Fig S25). Furthermore, we found that the ability to map cells with allelic imbalance information was weak, with the probability of mapping to a cell not exceeding 0.6 in the vast majority of cells (Fig S26).

	A	B	C
A	926	3	0
B	4	187	1
C	0	2	29

Table S1: Confusion matrix showing agreement between clonealign including allelic imbalance information (rows) vs without (columns).

4 Effect of cell cycle on clone assignment

To evaluate the effect of cell cycle on clone assignment, we computed cell cycle stage for the SA501 using the scran package (2), and used the probability that a cell was in G2M phase as a covariate for input to clonealign. We found highly concordant clone assignments between inference performed controlling for cell cycle as a covariate and inference performed not controlling for cell cycle (table S2).

5 Stability to random seeds

Clonealign uses a Monte-Carlo estimate of the ELBO during variational inference which is inherently random and dependent on the initializing seed. To test the robustness of clonealign to the seed we performed inference on the SA501 dataset using 10 different seeds. We found that over the 10 fits

	A	B	C
A	929	1	0
B	1	191	0
C	0	4	26

Table S2: Confusion matrix showing agreement between clonealign not controlling for cell cycle (rows) vs clonealign controlling for cell cycle (columns).

only 8 of the 1152 cells were assigned to different clones between runs (Fig S27a), and of these 8 cells, 6 were assigned a different clone in 1 fit only (Fig S27b).

6 Comparison to basic correlation method

We compared *clonealign* against a basic correlation method for assigning cells to clones. This method consists of the following steps:

1. Scale the columns (genes) of the log-count matrix to have mean 0 and standard deviation 1
2. Compute the Spearman correlation ρ_{nc} of the expression of cell n with the copy number of clone c
3. Set $z_n = \arg\max_c \rho_{nc}$

In the ideal limit that we get a noise-free measurement of expression that is perfectly correlated with copy number then this approach will work well.

We compared clonealign to this correlation method on the SA501 dataset. In the absence of a ground-truth labels for the clones of the scRNA-seq cells, we assess the methods using two heuristics: (1) the ability to recapitulate the clonal proportions observed in the single-cell DNA-seq data, and (2) the ability to separate expression of *XIST* between clones A and B-C, since we know *XIST* expression should be downregulated in A due to loss of the inactive X-chromosome.

The results can be seen in Fig S28. The clonal assignment using clonealign agrees much better with the single-cell DNA-seq than the clonal assignment using the basic correlation method, which underestimates the abundance of clone A by over 20% and underestimates the abundance of clone C by over 20%. Similarly, clonealign separates expression of *XIST* between clones A and B-C, with only a few cells in A expressing *XIST*. In comparison, the correlation method assigns only a few cells to A that express *XIST*, but also assigns many cells to clones B and C that do not express *XIST*, contrary to the known biology. We quantified this dependency by treating the expression of *XIST* as a "ground truth" of the membership of clones B and C, and then treating clonealign/correlation method as a classifier calculated the F1 score for each. This gave an F1 for clonealign of 0.958 and an F1 score of 0.845 for the correlation method, demonstrating the advantage of the full noise model implemented in clonealign on the real single-cell data.

We further included the correlation method in the simulations concerning robustness to the (latent) proportion of genes that have a copy-number expression (dosage) relationship. We choose this simulation as in this situation both correlation and clonealign are mis-specified with respect to the simulation model (clonealign does not know which genes are simulated as having a copy-number expression dependency and "believes" they all do), as in all the other simulations we effectively simulate from the clonealign model which would unfairly advantage clonealign.

We see that clonealign is much more robust to the underlying proportion of genes that exhibit a copy-number dosage effect (Fig S29). As this proportion is increased to 1 the performance of the

basic correlation method almost matches clonealign, which is to be expected as at this level the transcriptomes and genomes are highly correlated. The point is that in real data we expect this correlation to be far less than perfect due to measurement error and transcriptional regulation, in which case the bespoke statistical model of clonealign performs best. We note that clonealign also accounts for both fixed effects (e.g. cell cycle as pointed out by the reviewer below) and random effects (ie accounts for more variation in the expression data than that explained by copy number alone).

References

[1] Zhang, M.J., Ntranos, V., Tse, D.: One read per cell per gene is optimal for single-cell rna-seq (2018)

[2] Lun, A.T., McCarthy, D.J., Marioni, J.C.: A step-by-step workflow for low-level analysis of single-cell rna-seq data with bioconductor. F1000Research **5** (2016)
