# Supplementary material for: clonealign: statistical integration of independent single-cell RNA and DNA sequencing data from human cancers
Source: Genome Biol. 2019 Mar 12;20:54. doi: 10.1186/s13059-019-1645-z (PMC6417140; doi:10.1186/s13059-019-1645-z)
Supplement: Supplementary file 2 — Supplementary figures. (PDF 6381 kb) [file 13059_2019_1645_MOESM2_ESM.pdf]

Additional file 2: Supplementary Figures for "clonealign: statistical integration of independent single-cell RNA & DNA-seq from human cancers"

January 30, 2019

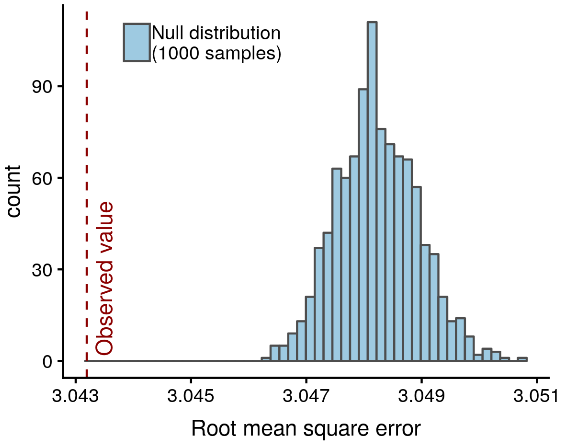

Figure S1: Distribution of root mean square error in predicting the expression of genes on held out chromosomes (8 & 18) for SA501 under random repeated permutation of clone assignments (light blue) compared to the observed error under clonealign assignments (red dashed arrow). This demonstrates the observed error is significantly less than is observed at random ($p < 10^{-3}$).

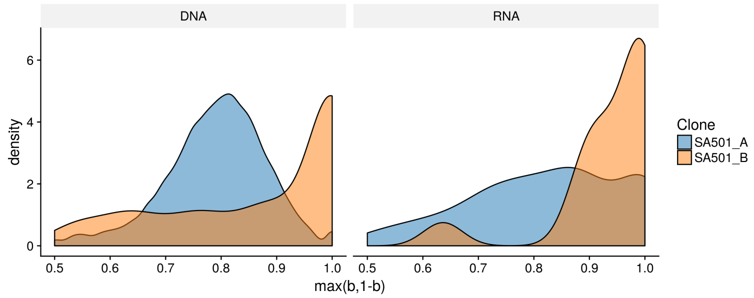

Figure S2: The b allele frequency for 5.8e7 on-wards on chromosome 18 demonstrates a reduction in clone B for both scRNA-seq and scDNA-seq. The region was called LOH by TitanCNA in clone B for both scRNA-seq and scDNA-seq.

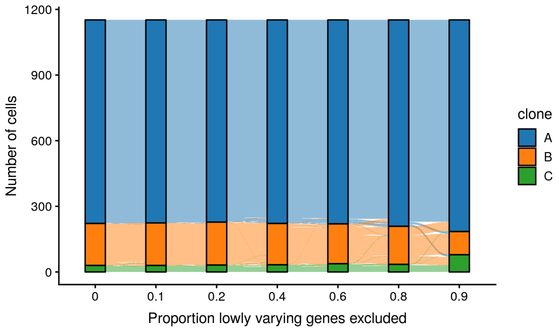

Figure S3: Robustness of clone assignments to gene selection in SA501X2B. At 0.1, only the top 90% most variable genes are retained, while at 0.2 the top 80% are retained, etc.

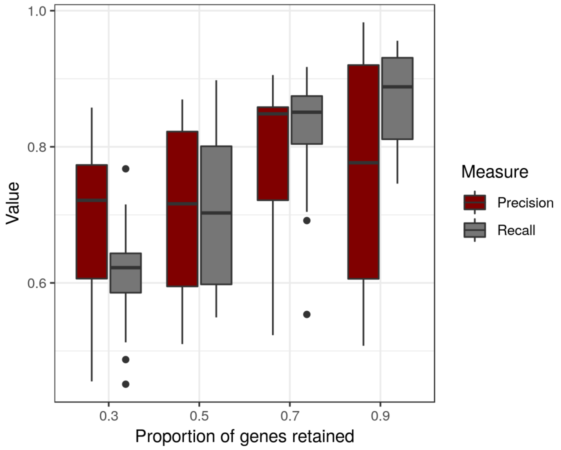

Figure S4: Robustness of clone assignments to gene selection in SA501X2B. Assuming the assignments computed using the full gene set are the "truth", the precision and recall is calculated for clone assignments using only a subset of highly variable genes. At 0.1, only the top 90% most variable genes are retained, while at 0.2 the top 80% are retained, etc.

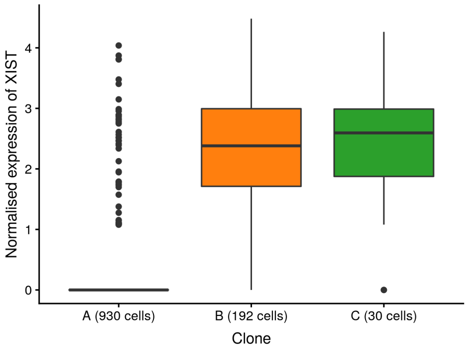

Figure S5: Log counts-per-million expression of *XIST* in clones A, B, and C in the SA501X2B breast cancer xenograft. Clone A is defined by a loss of X event, so downregulation of *XIST* in A implies the inactive X chromosome was lost.

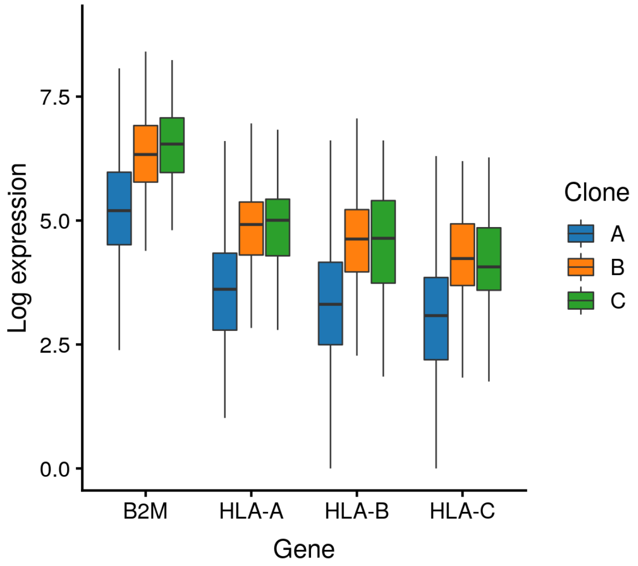

Figure S6: Log counts-per-million expression of MHC class-I genes between clones A, B & C in SA501X2B.

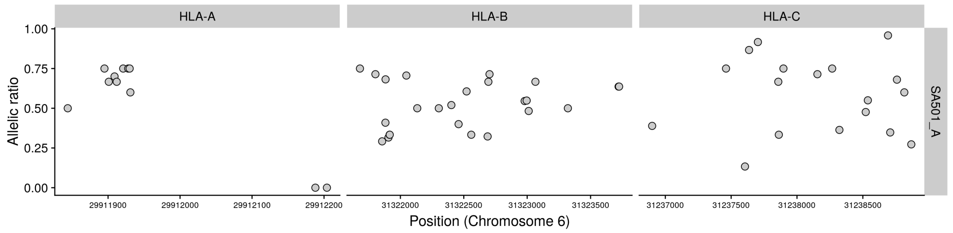

Figure S7: Allelic ratio on germline heterozygous locations for the *HLA-A*, *HLA-B*, and *HLA-C* genes. The samples lacked depth in both clone B and the *B2M* gene to call variants.

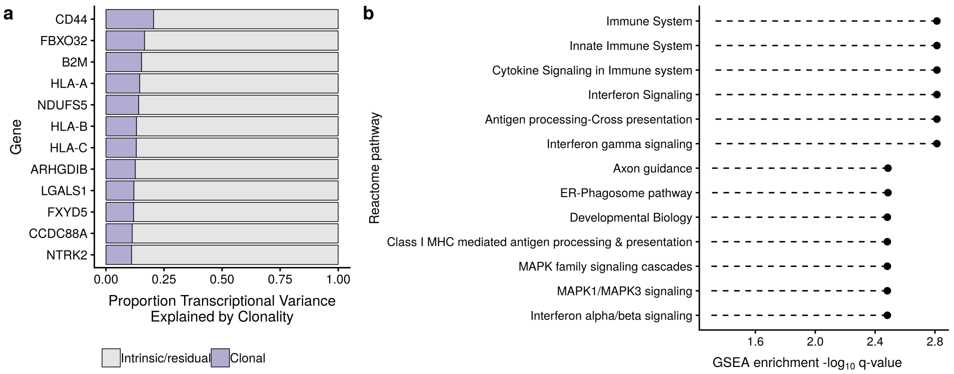

Figure S8: **a** The top genes in terms of proportion of transcriptional variance explained by clonality. **b** A gene set enrichment analysis of Reactome pathways on the genes ranked by proportion of transcriptional variance explained by clonality highlights clone-specific expression patterns of immune pathways, including MHC class-I mediated antigen presentation.

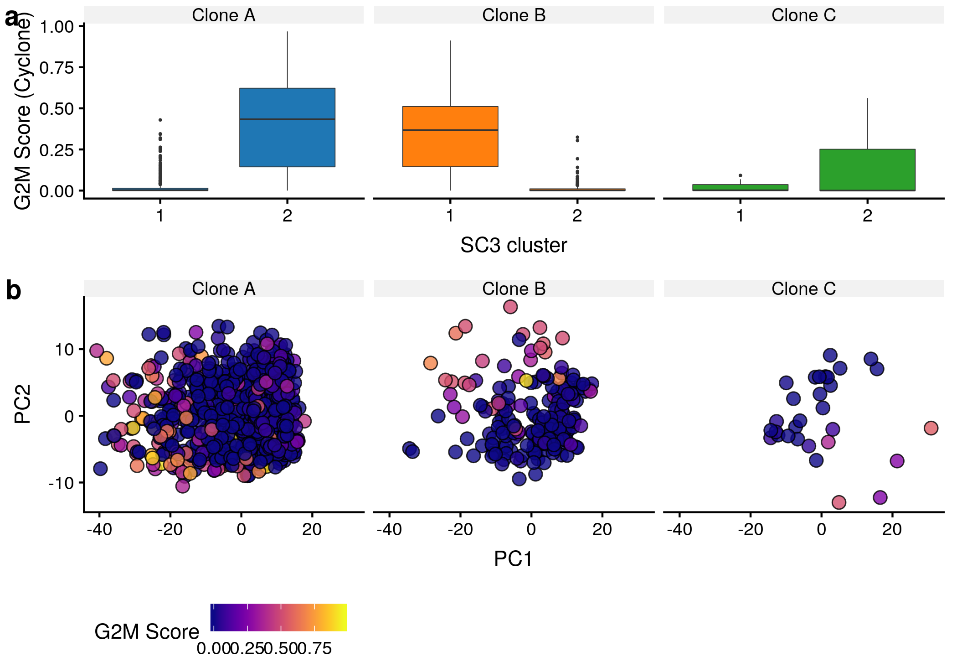

Figure S9: **a** G2M score (as called with Cyclone) compared to the SC3 cluster for each of the clones. **b** PCA plots of the three clones separately coloured by G2M score.

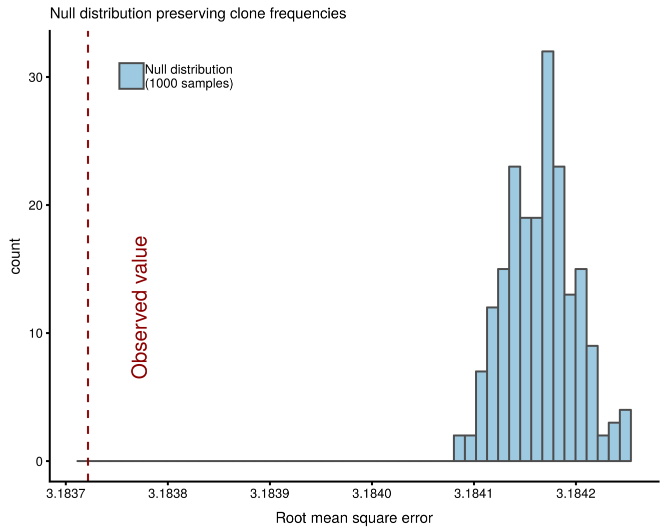

Figure S10: Distribution of root mean square error in predicting the expression of genes on held out chromosomes (1 & 14) for TOV2295R under random repeated permutation of clone assignments (light blue) compared to the observed error under clonealign assignments (red dashed arrow). This demonstrates the observed error is significantly less than is observed at random ($p < 10^{-3}$).

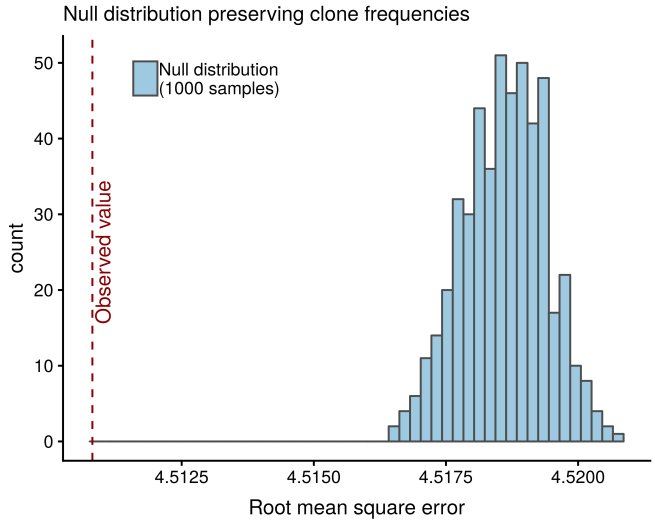

Figure S11: Distribution of root mean square error in predicting the expression of genes on held out chromosome (8) for OV2295R under random repeated permutation of clone assignments (light blue) compared to the observed error under clonealign assignments (red dashed arrow). This demonstrates the observed error is significantly less than is observed at random ($p < 10^{-3}$).

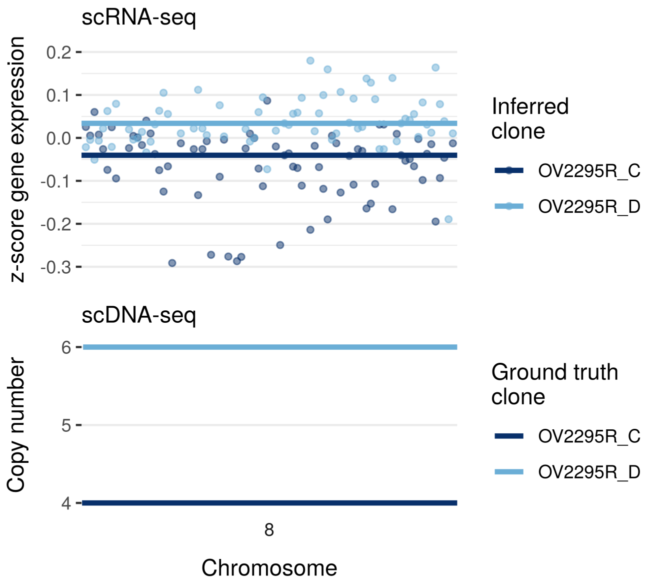

Figure S12: Gene expression and copy number on a held out segment (chromosome 8) for the OV2295R analysis.

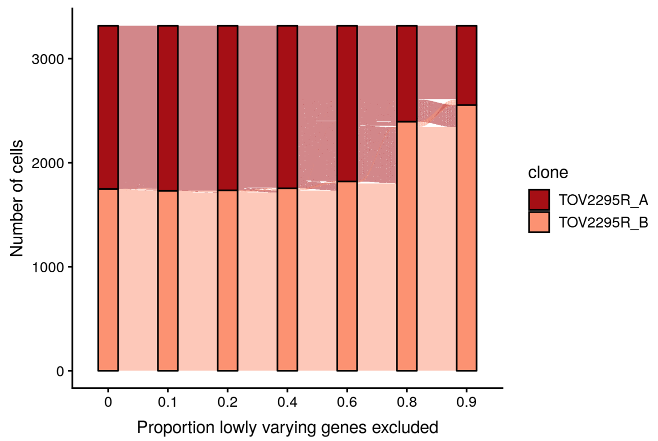

Figure S13: Robustness of clone assignments to gene selection in TOV2295R. At 0.1, only the top 90% most variable genes are retained, while at 0.2 the top 80% are retained, etc.

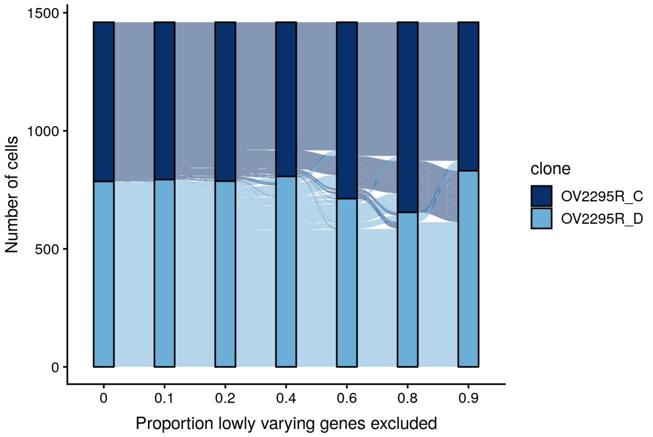

Figure S14: Robustness of clone assignments to gene selection in OV2295R. At 0.1, only the top 90% most variable genes are retained, while at 0.2 the top 80% are retained, etc.

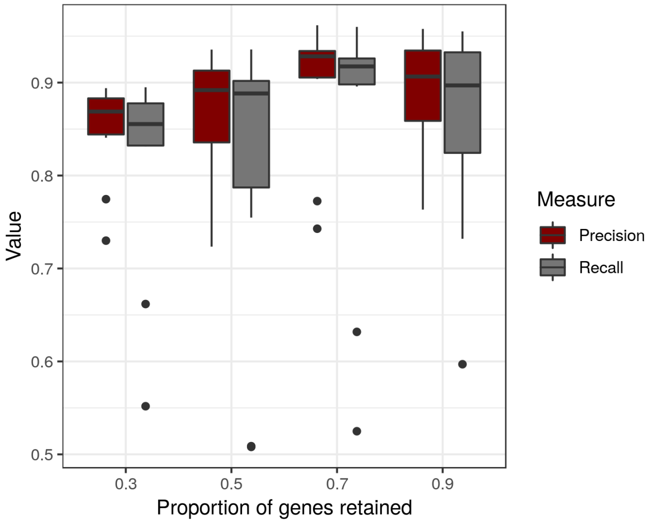

Figure S15: Robustness of clone assignments to gene selection in TOV2295R. Assuming the assignments computed using the full gene set are the "truth", the precision and recall is calculated for clone assignments using a random set of genes sampled from the transcriptome.

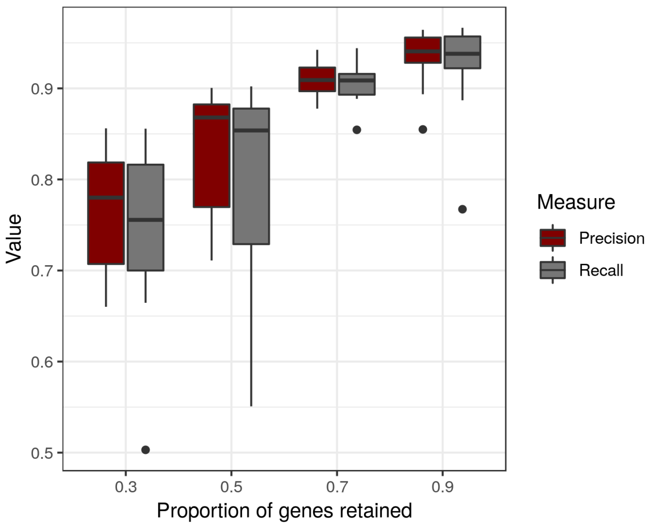

Figure S16: Robustness of clone assignments to gene selection in OV2295R. Assuming the assignments computed using the full gene set are the "truth", the precision and recall is calculated for clone assignments using a random set of genes sampled from the transcriptome.

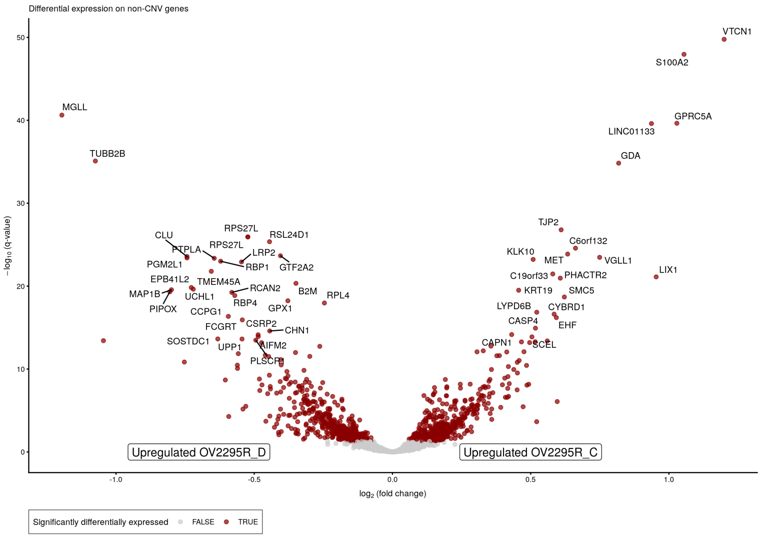

Figure S17: Volcano plot of differential expression between OV2295R clones OV2295R_C and OV2295R_D.

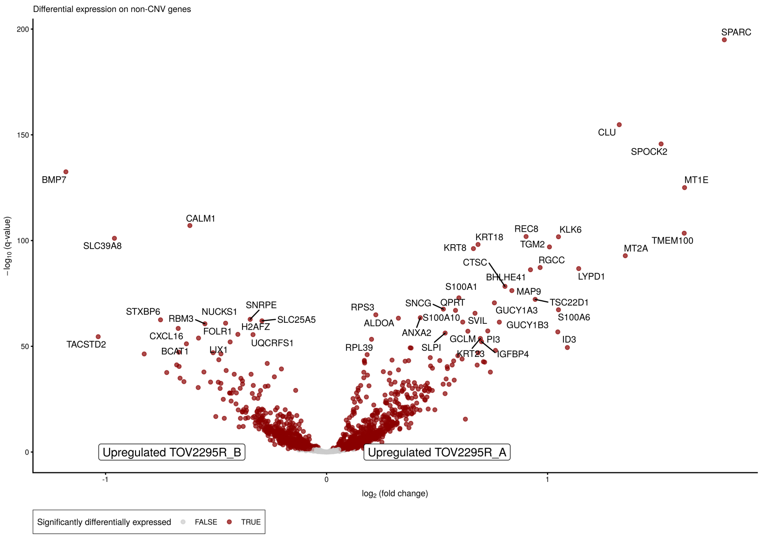

Figure S18: Volcano plot of differential expression between TOV2295R clones TOV2295R_A and TOV2295R_B.

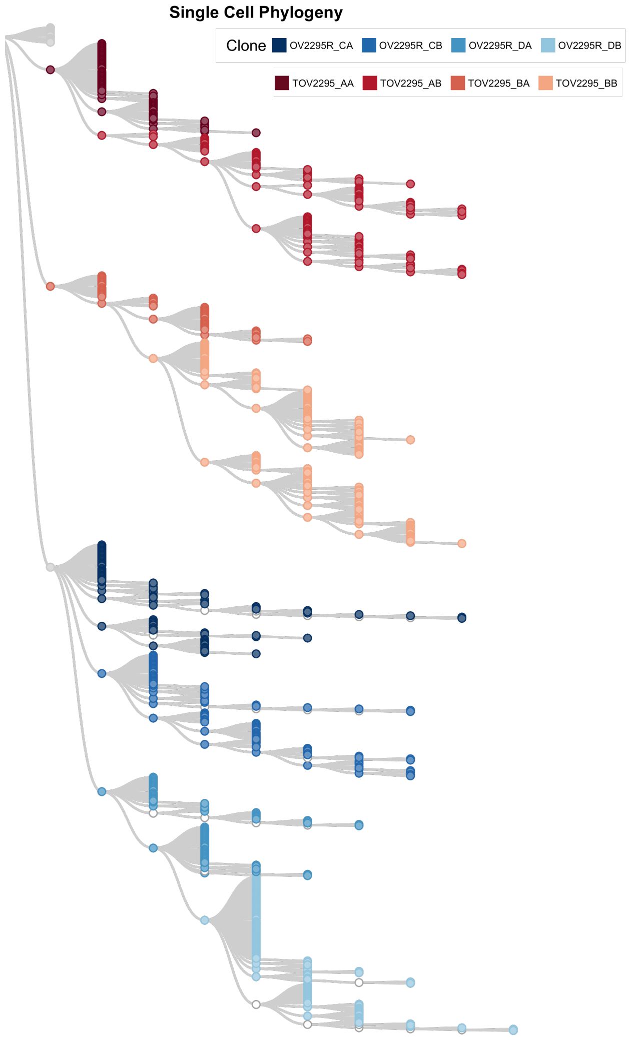

Figure S19: Phylogenty for (T)OV2295 when split into eight clades (clones).

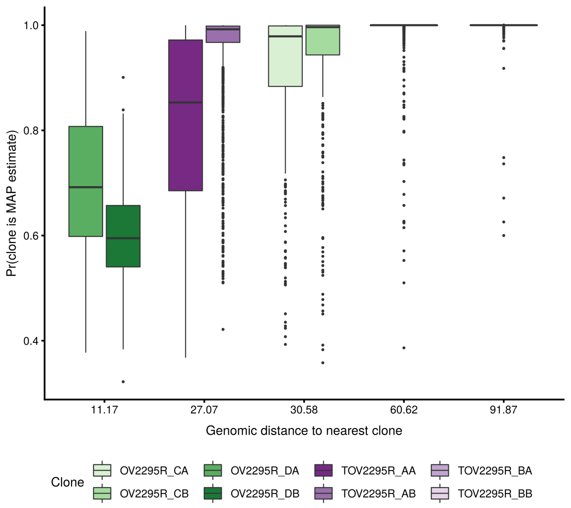

Figure S20: The maximum likelihood probability for a cell to be assigned to a clone as a function of the genomic distance (euclidean distance in copy number space) to the nearest clone. The more distinct clones are, the more certainty in clonal assignment, while for clones that are very close in copy number space the model assigns uncertainty to the assignment in RNA-space.

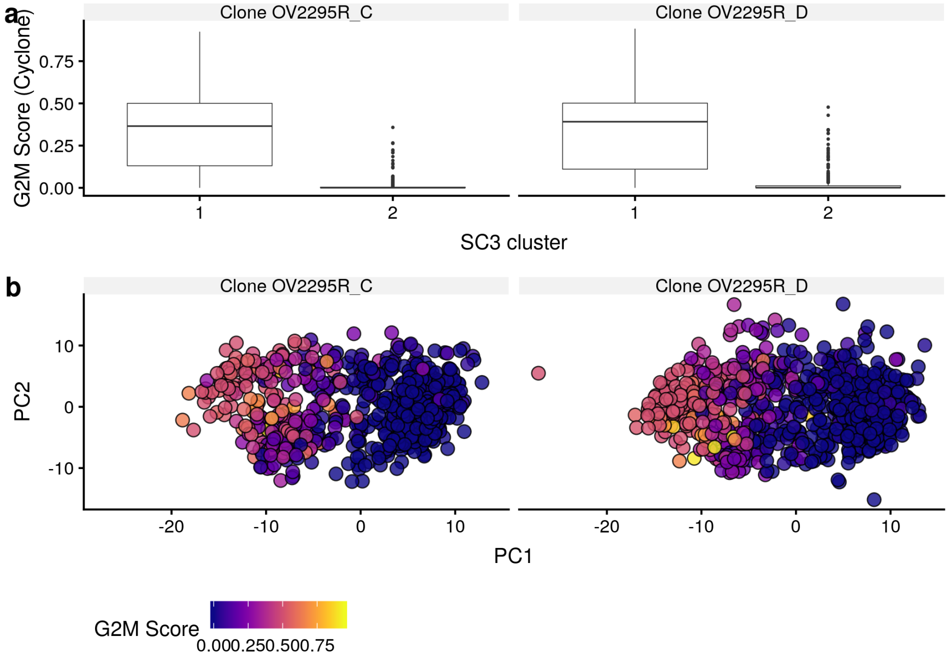

Figure S21: **a** G2M score (as called with Cyclone) compared to the SC3 cluster for each of the clones from OV2295R. **b** PCA plots of the three clones separately coloured by G2M score.

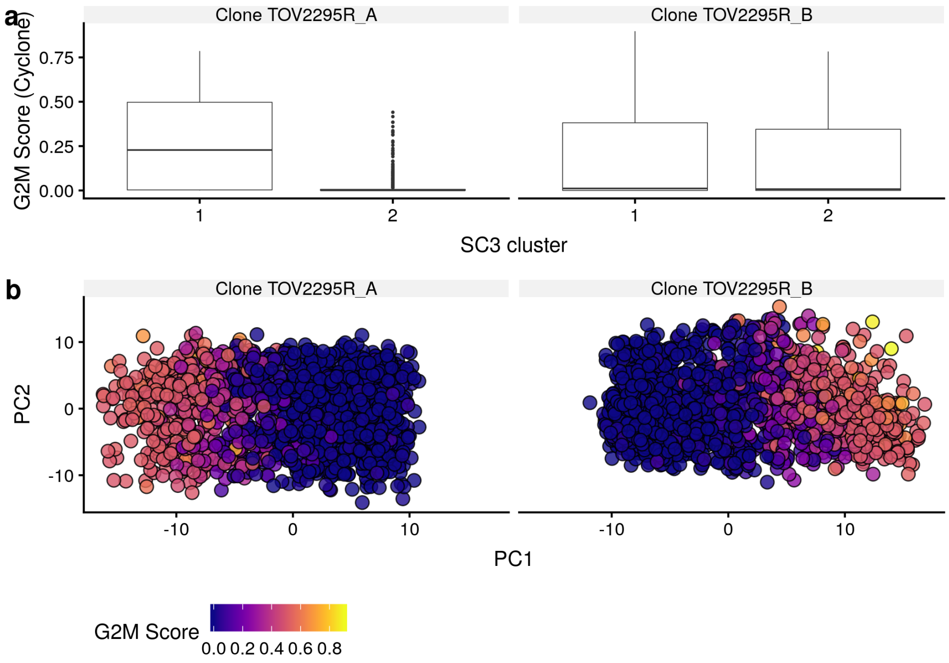

Figure S22: **a** G2M score (as called with Cyclone) compared to the SC3 cluster for each of the clones from TOV2295R. **b** PCA plots of the three clones separately coloured by G2M score.

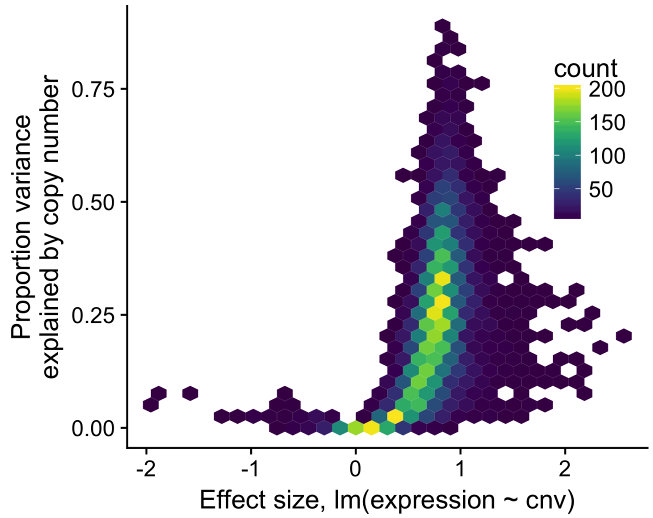

Figure S23: The effect size and reported R^2 of a linear model regressing the log counts on the $\log R$ (copy number) values in the BRCA TCGA cohort.

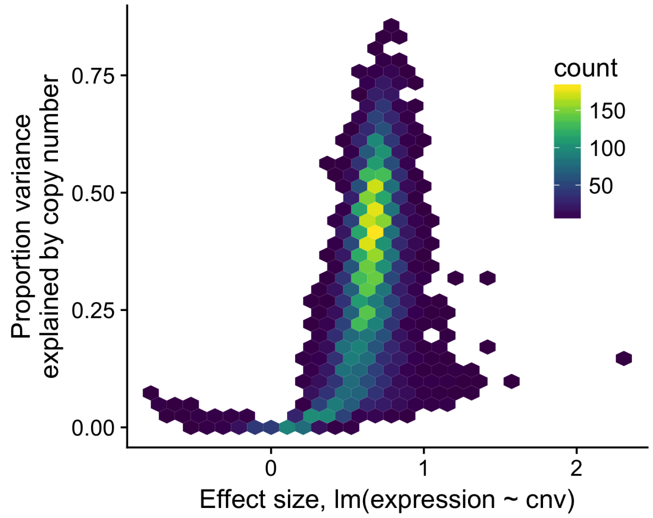

Figure S24: The effect size and reported R^2 of a linear model regressing the log counts on the $\log R$ (copy number) values in the OV TCGA cohort.

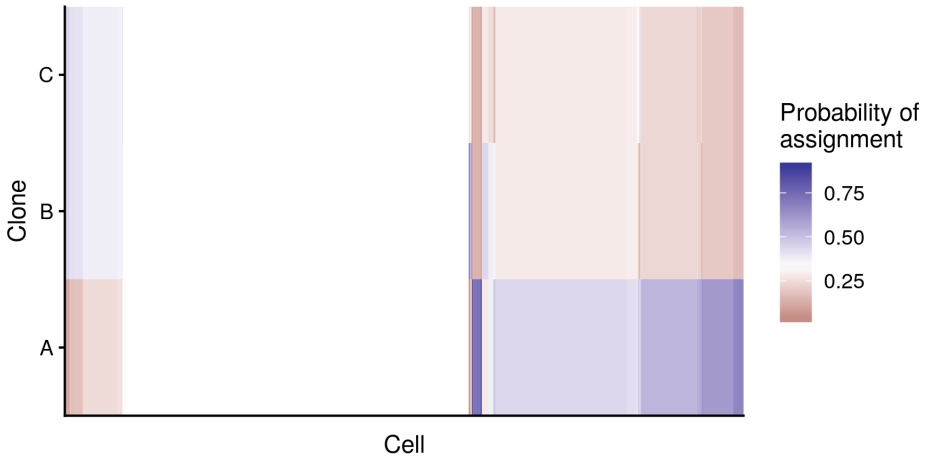

Figure S25: Assignment probabilities using allele specific info for SA501 only

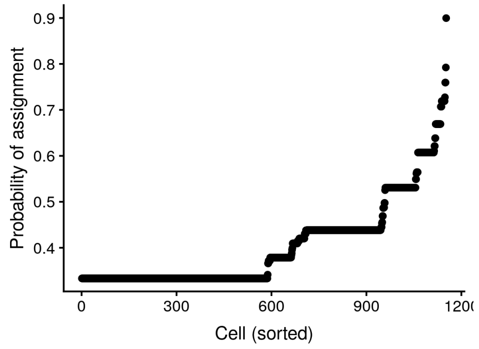

Figure S26: Maximium assignment probability of each cell for SA501 dataset.

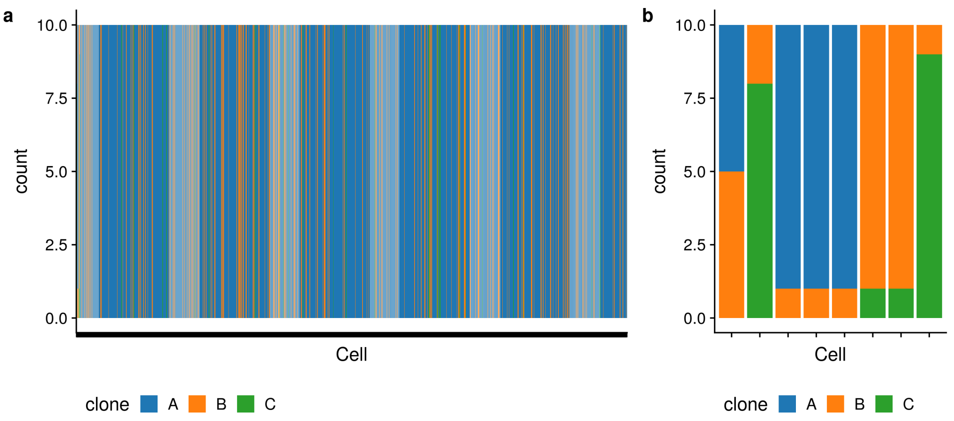

Figure S27: **a** Number of times each cell was assigned to a clone over 10 random seeds. **b** The 8/1152 cells assigned to different clones over the 10 random seeds.

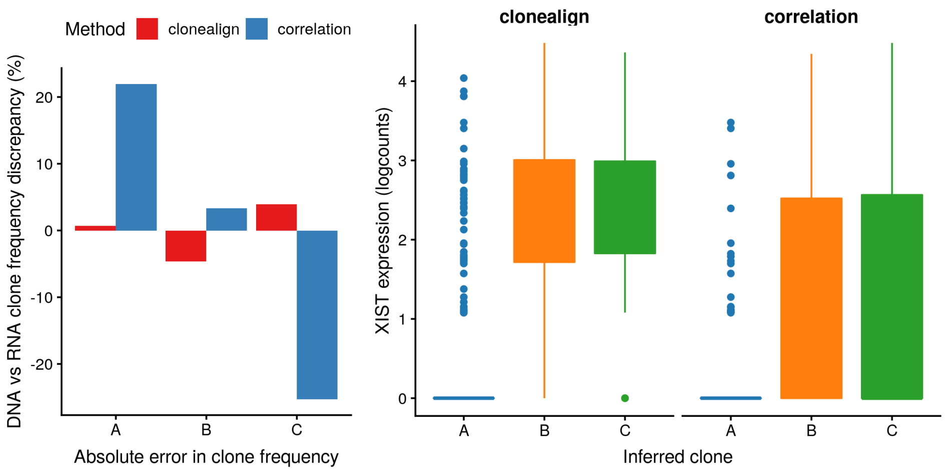

Figure S28: Comparison to a basic correlation method. (A) Difference in clonal frequencies of clones inferred from scRNA-seq compared to ground truth (scDNA-seq) using both clonealign and a basic correlation method. (B) Expression of *XIST* across the three clones depending on clonal inference method used.

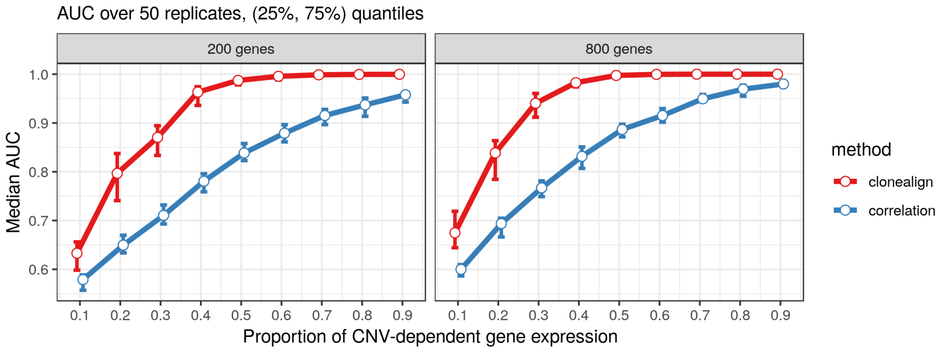

Figure S29: AUC of clonal assignment accuracy as a function of the number of genes exhibiting a copy number dosage effect for 200 and 800 genes and the clonealign and basic correlation methods.

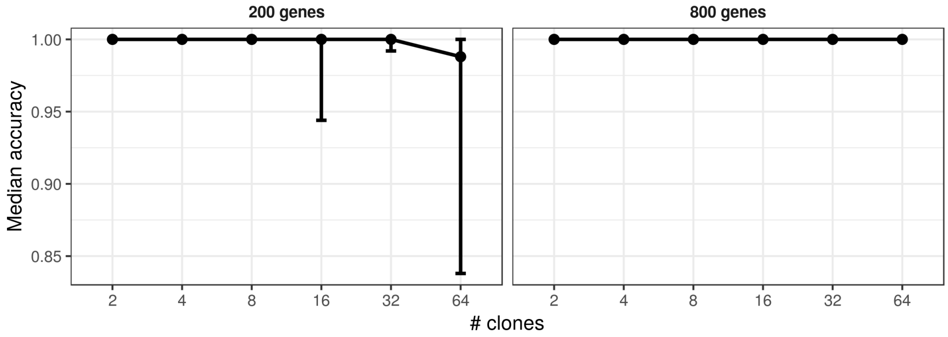

Figure S30: Accuracy of clonealign as the number of clones increases.

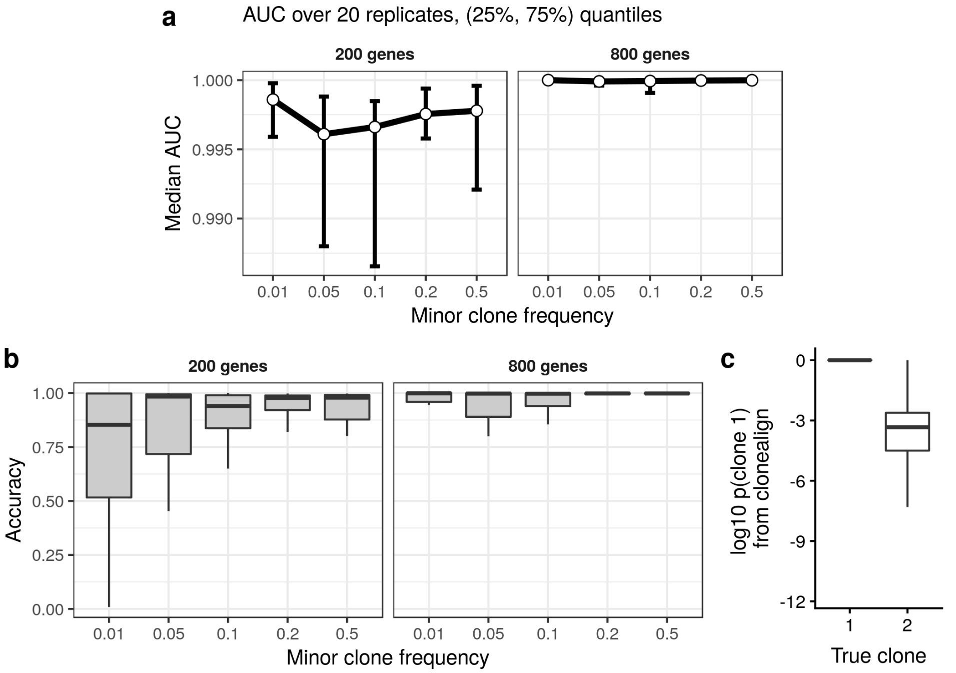

Figure S31: Robustness of clonealign to minor clone frequency. **a** As measured by area under receiver operator curve (AUC) **b** Accuracy (proportion of clones correctly identified) **c** For very minor clone frequencies, the model probabilities can be miscalibrated, leading to a high AUC but low accuracy.

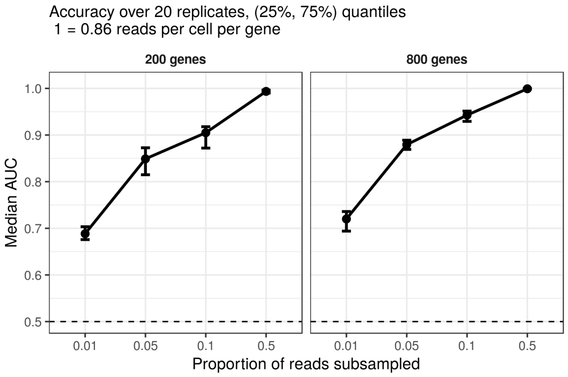

Figure S32: Clonal inference performance as a function of quality (through subsampling reads).

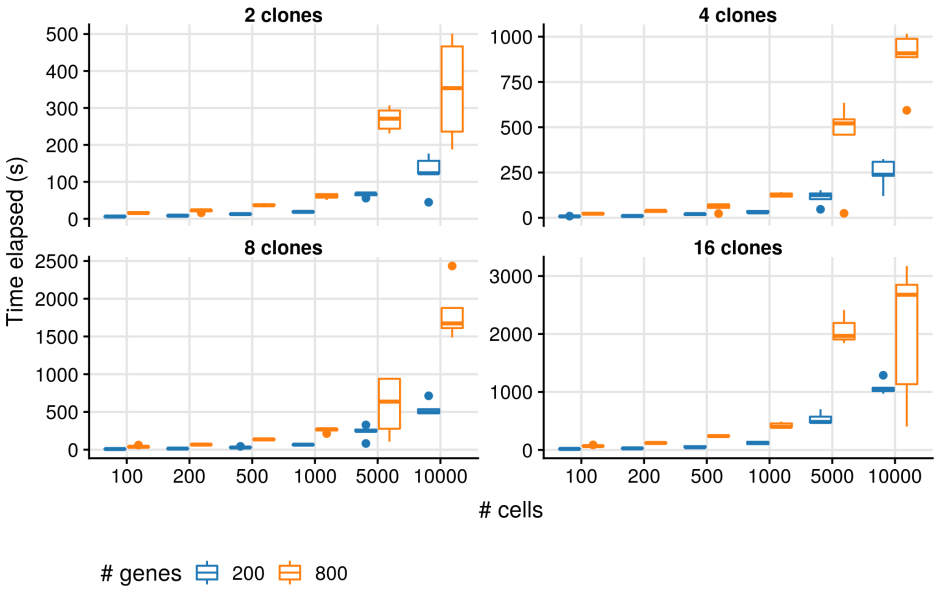

Figure S33: Performance benchmarking of clonealign.
